# Large-area thin-film synthesis of photoactive Cu_3_PS_4_ thiophosphate semiconductor with 0–14 pH stability range

**DOI:** 10.1039/d5sc05882a

**Published:** 2025-10-14

**Authors:** Lena A. Mittmann, Javier Sanz Rodrigo, Eugène Bertin, Giulia Dalmonte, Jean-Claude Grivel, Ivano E. Castelli, Andrea Crovetto

**Affiliations:** a National Centre for Nano Fabrication and Characterization (DTU Nanolab), Technical University of Denmark 2800 Kongens Lyngby Denmark mittma@dtu.dk ancro@dtu.dk; b Department of Energy Conversion and Storage (DTU Energy), Technical University of Denmark 2800 Kongens Lyngby Denmark

## Abstract

Metal phosphosulfide materials have sparked growing interest due to their wide range of properties and applications. Despite a sizable presence in the bulk materials synthesis literature, reports of phosphosulfide thin films are extremely scarce. This may be due to the hazardous, volatile, and corrosive nature of many phosphorus and sulfur precursors, combined with the high sulfur chemical potential needed to incorporate large amounts of this volatile element in the (PS_4_)^3−^ and (P_2_S_6_)^4−^ thiophosphate anions that are common in this material family. To overcome these limitations, we introduce directional-and-diffuse multi-anion reactive sputtering (DADMARS). DADMARS uniquely combines sputtered metal sources, reactive gas, and a thermally cracked evaporated nonmetal source with a high chemical potential to gain access to challenging multi-anion chemistries in thin-film form. In this study, we employ Cu, PH_3_, and S_*x*_ as the sputtered, gaseous, and evaporated sources to deposit polycrystalline Cu_3_PS_4_ thin-film thiophosphate semiconductors with the narrowest X-ray diffraction peaks recorded for a phosphosulfide thin film. Single-phase Cu_3_PS_4_ can be grown over relatively large areas (8 × 8 cm^2^ and extendable) at temperatures down to 375 °C, which is lower than for similar sulfide semiconductors CuInS_2_ and Cu_2_ZnSnS_4_. This suggests potential compatibility with established device fabrication processes. Cu_3_PS_4_ thin films exhibit remarkable environmental, radiation, and chemical stability, with negligible etch rates in the 0–14 pH range. Thin-film Cu_3_PS_4_ is a p-type semiconductor with a bandgap of 2.3–2.5 eV, strong light absorption, and detectable photoluminescence at room temperature. This combination of stability and optoelectronic properties positions Cu_3_PS_4_ as a promising earth-abundant semiconductor for photoelectrochemical applications.

## Abstract 2

We develop directional-and-diffuse multi-anion reactive sputtering to demonstrate the first large-area thin-film growth of a crystalline metal thiophosphate.

## Graphical abstract

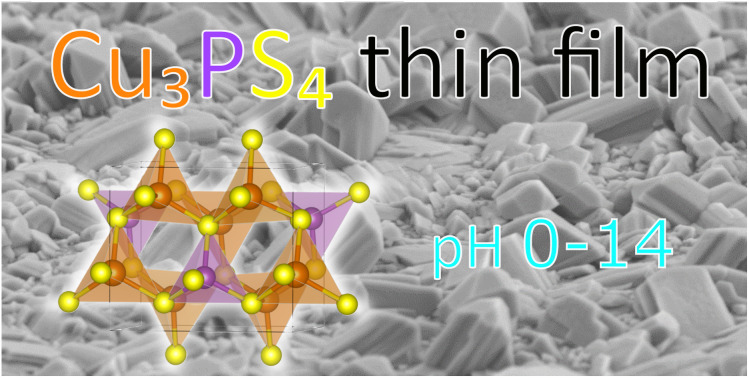

## 1. Introduction

Inorganic compounds containing phosphorus, sulfur and at least one metal (phosphosulfides) are relatively well explored in the form of bulk crystal or powder samples, with over 250 reported compositions in the Inorganic Crystal Structure Database (ICSD).^1^ Most – although by no means all – of these known compounds are thiophosphates, the sulfur analogues of phosphates. Common thiophosphate compositions are M_*x*_PS_4_, M_*x*_PS_3_, and M_*x*_P_2_S_7_, where M is a metal and phosphorus is in the +5 or +4 oxidation state. Recently, synthesis of thiophosphate nanoparticles,^2,3^ thick films from slurries of bulk powders,^4,5^ and 2D thiophosphates near the atomically-thin limit (*via* single-crystal exfoliation^6^ or chemical vapor deposition^7,8^) has been reported. Yet, well-characterized thin-film phosphosulfides are almost non-existing, with the known reports (less than ten)^2,3,9–15^ exhibiting amorphous^10–14^ or uncoalesced films,^2,3^ solid solutions rather than unique compounds,^15^ or questionable prospects for the deposition technique to be extended to other phosphosulfides.^9,10^ 2D material growth has so far resulted in isolated flakes tens of μm in size.^7,8^ None of the previous reports of thin-film growth has demonstrated continuous large-area deposition, high crystalline quality with grains larger than a μm, or extensive characterization.

The lack of thin-film phosphosulfide materials research hinders at least three important developments: (i) applications where the thin-film form is necessary or highly desirable (*e.g.*, photovoltaics, solid-state lighting, photoelectrochemistry, microelectronics, quantum technology, and any kind of on-chip application); (ii) property measurements where the material should be continuous over a sufficiently large area (*e.g.*, light absorption coefficient and some electrical measurements); and (iii) high-throughput determination of composition–structure–property relationships as a function of growth conditions.

In this contribution, we propose a thin-film growth strategy combining a sputtered metal vapor, a cracked evaporated sulfur beam,^16–18^ and a diffuse source of phosphorus (PH_3_).^19–23^ This growth technique can be broadly classified as reactive sputtering, but it is made unique by the different nature of the two reactive sources, one being a diffuse gas and the other a directional beam. We refer to it as directional-and-diffuse multi-anion reactive sputtering (DADMARS). Since the metal, phosphorus, and sulfur sources are independent of each other and spatially separated, this deposition technique has two major advantages. First, it can be used to attempt growth of any phosphosulfide compound regardless of the metal (or metals) and of elemental composition. Second, it is compatible with both high-throughput combinatorial research (due to the directionality of the metal and sulfur vapors) and large-area uniform deposition. The highly tunable chemical potential of the cracked sulfur beam enables us to access compounds with high sulfur content, such as thiophosphates, where a diffuse H_2_S source with lower chemical potential may not be sufficient.^16^ We previously suggested this deposition strategy in a perspective article,^1^ and now we report the first example of a phosphosulfide thin film grown by this method.

The proposed technique is applied to grow thiophosphate Cu_3_PS_4_ films. Cu_3_PS_4_ is a p-type semiconductor first synthesized in bulk form in 1894.^24^ It has a band gap in the visible range^25–27^ and preliminary but incomplete indications of photoactivity,^3,25,26^ in addition to high chemical stability.^24^ The naturally occurring isostructural compound (enargite Cu_3_AsS_4_) has received more detailed attention as a material for optoelectronics/photoelectrochemistry^28–30^ with significant coalescence and grain growth in nanoparticle-coated thin films^29^ and encouraging non-radiative carrier lifetimes in the ns range.^30^ On the other hand, detailed investigations on the optoelectronic properties of Cu_3_PS_4_ are missing, with a lack of absorption coefficient, photoluminescence spectra, carrier lifetimes, and reported difficulties in carrier mobility measurements due to the low density of pelletized bulk samples.^26^ We hypothesize that the paucity of reports about the electrical and optical properties of Cu_3_PS_4_, their dependence on growth conditions, and optoelectronic/photoelectrochemical applications is due to the lack of a suitable technique to deposit phosphosulfide films in general (points (i–iii) above).

In fact, the main focus of research on Cu_3_PS_4_ has been within applications for which thin-film samples are not necessary, *e.g.*, as an electrode for Li-ion,^31,32^ Mg-ion,^33^ and Na-ion batteries,^34,35^ electrocatalysis,^36,37^ second harmonic generation,^38,39^ and ammonia sensing.^40^ Cu_3_PS_4_ has been prepared by solid-state reactions,^24,41^ chemical vapor transport,^42^ ball milling^26,35^ and solvent based synthesis.^2,3^ These methods yield Cu_3_PS_4_ in crystal, powder, or nanoparticle form. Unlike the case of Cu_3_AsS_4_, the only reported attempts to deposit Cu_3_PS_4_ films from nanoparticles did not result in nanoparticle coalescence and grain growth.^2,3^

Employing the DADMARS technique, we prepared continuous single-phase Cu_3_PS_4_ thin films with substantial grain growth at temperatures below 500 °C. The Cu_3_PS_4_ films show remarkable stability in air and chemical resistance across the full pH range from acidic (pH = 0) to basic (pH = 14). Beyond the synthesis, we combine detailed experimental characterization with electronic structure calculations *via* density functional theory (DFT) to provide insights into the material's bonding and optoelectronic behavior. Overall, Cu_3_PS_4_ appears to be a chemically resilient and promising candidate for future applications involving light and electricity.

## 2. Experimental details

### 2.1 Equipment

**Fig. 1:**
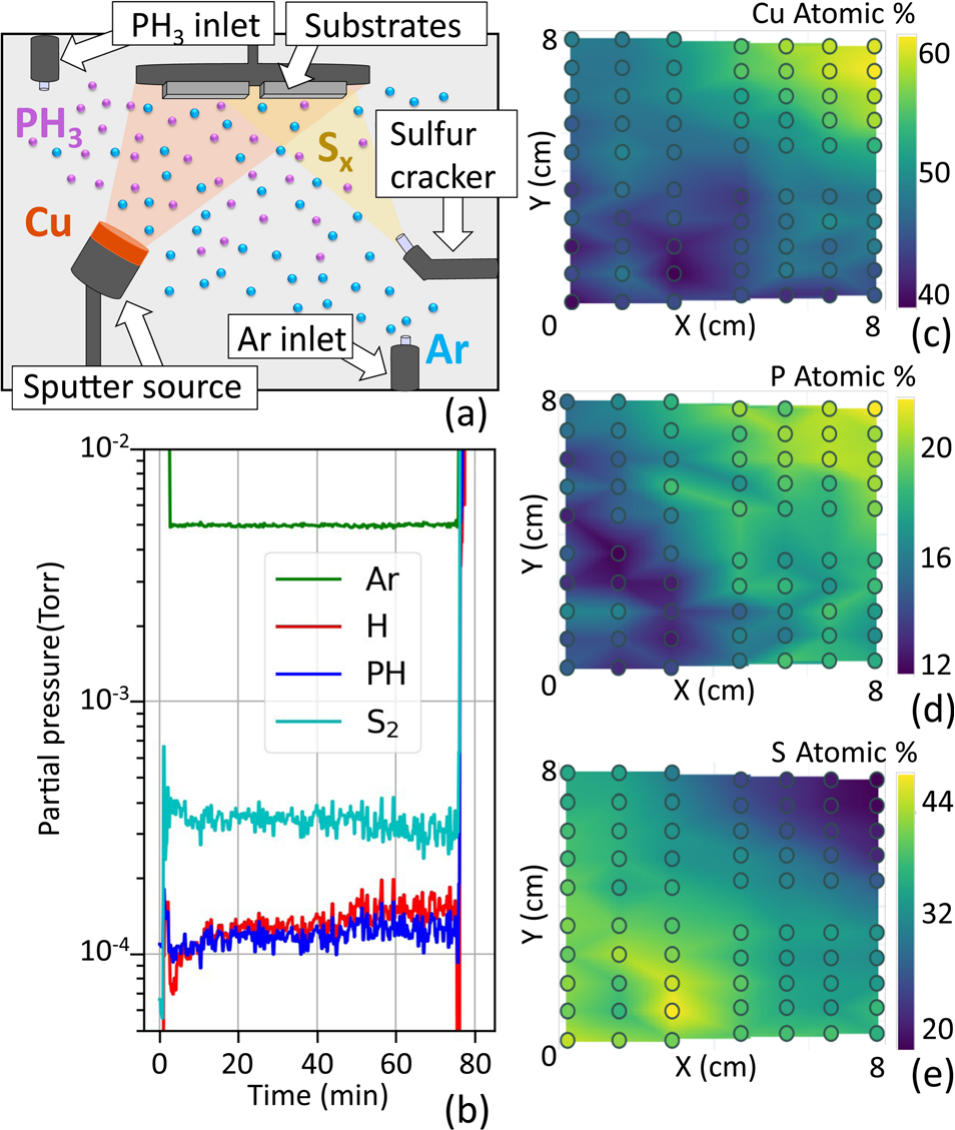
DADMARS synthesis apparatus: (a) scheme of the reactive sputter chamber combining a Cu target, a directional S source and two separate gas inlets, one for the reactive gases like PH_3_ and one for Ar. (b) Partial pressures of the gases tracked by OES during deposition of Cu_3_PS_4_ (c–e) EDX composition maps of Cu (c), P (d), and S (e) in a combinatorial film deposited with this setup.

The film deposition apparatus used in this work is a custom reactive sputtering chamber (Kurt J. Lesker) with three magnetron sources, an S-cracker and separate gas inlets for the inert and reactive gases (Fig. 1a). The S-cracker (Nano4Energy/Gencoa) is a directional sulfur beam source, where elemental sulfur is first heated in an effusion cell to reach its equilibrium vapor pressure. It is then released towards the main deposition chamber *via* a pulsed valve and heated further to crack the S_8_ rings of low-temperature sulfur into smaller, more reactive species. The sulfur vapor flux at the substrate is controlled by the duty cycle of the pulsed valve. The combination of sputter targets and reactive species from both gaseous sources (PH_3_) and evaporated sources (S_*x*_) gives flexibility for the synthesis of challenging materials. The setup enables controlled compositional and thickness gradients by adjustment of the angles and positions of the sputter targets and the S-cracker nozzle with respect to the stationary substrates. The deposition chamber is directly connected to an actively purified N_2_-filled glovebox (LC Technology) so samples can be retrieved and stored without air exposure.

### 2.2 Synthesis

The Cu_3_PS_4_ thin-film samples were synthesized by directional-and-diffuse multi-anion reactive sputtering (DADMARS) using a metallic Cu target in a magnetron source, reactive PH_3_ gas, and a cracked sulfur beam. RF power was supplied to the Cu target at 25 W which resulted in a target DC self-bias between 175 and 200 V during the deposition step with the reactive sources turned on. The pressure during the deposition was controlled to be 5 mTorr under 3.5 sccm of PH_3_ and 131.5 sccm of Ar. The S-cracker was run with a cracking temperature of 400 °C, where S_2_ is expected to be the dominant species at these pressures.^43^ The background sulfur partial pressure (away from the direct sulfur beam) was adjusted to be between 0.2 and 0.5 mTorr, as measured by an ion gauge before introducing the other gases. The partial pressures of all gases during deposition were monitored in real time by optical emission spectroscopy (OES, Fig. 1b) using a remote plasma generator (OPTIX, Gencoa). The PH_3_ partial pressure (0.10–0.14 mTorr) is rather stable over an hour (Fig. 1b) and close to the value expected from the relative PH_3_ flow rate (0.13 mTorr). The OES-measured sulfur partial pressure (0.3–0.4 mTorr) is also stable and consistent with the ion gauge value (Fig. 1b).

The deposition rate, determined by dividing the measured sample thickness by the deposition time, was between 0.5 and 2 Å s^−1^ depending on the position on the substrate. The highest rates were obtained at positions closest to the Cu target. The deposition time varied between 25 and 100 min, so film thicknesses were in the hundreds of nm range. The substrate holder was positioned face down above the targets in the chamber. A combination of substrates was mounted to cover an area of about 8 × 8 cm^2^ (Fig. 1). Details on the substrate materials used for each measurement are provided in the SI (Tables S1 and S2). During deposition, the substrate holder was kept at floating potential and at a temperature of 465 °C.

### 2.3 Characterization

The bulk, depth-averaged elemental composition and thickness of the thin films was determined by energy dispersive X-ray spectroscopy (EDX) using a FEI Quanta FEG 250 equipped with an Oxford Instruments EDX detector. The elemental composition was mapped as a function of position throughout the combinatorial samples using the LayerProbe software (Oxford Instruments), resulting in composition maps such as the ones shown in Fig. 1c–e. The LayerProbe software calculates a self-consistent film composition and mass thickness by fitting EDX spectra containing both film- and substrate features. The film thickness was calculated from the mass thickness assuming the density of bulk Cu_3_PS_4_ (4.33 g cm^−3^).^38^

Scanning electron microscopy (SEM) images were recorded with a Zeiss Gemini SEM 560 to inspect the thin-film morphology.

The surface composition and chemical states of the elements were determined by X-ray photoelectron spectroscopy (XPS) using an Al-Kα source. The valence band onset was measured by ultraviolet photoelectron spectroscopy (UPS) using a He–I source. Both measurements were conducted in a Thermo Fischer Nexsa system. The samples were transferred between the glovebox and the measurement instrument in a dedicated vacuum transfer module to avoid ambient air exposure and surface oxidation. Therefore, XPS and UPS measurements were conducted on the native sample surface without *in situ* ion-beam sputtering.^44^

X-ray diffraction (XRD) measurements were performed to probe crystal structure, phase purity, and stability in air, acid, and base. A Rigaku SmartLab diffractometer in the parallel beam *θ*–2*θ* geometry with a rotating Cu-Kα source, focusing optics and a 2D detector was employed.

The vibrational fingerprints of the films were investigated with Raman spectroscopy, using a Renishaw InVia confocal Raman microscope with a 785 nm excitation wavelength.

An Agilent Cary 7000 instrument with an integrating sphere was used to measure direct and diffuse UV-Vis-NIR transmission and reflection spectra for the determination of the absorption coefficient of the film. Photoluminescence (PL) properties were measured using a custom setup with a 405 nm laser for excitation.

Different substrate materials (crystalline Si, fused silica, and soda lime glass) were chosen to accommodate the requirements of each characterization technique. Composition, crystal structure and morphology were examined across all types of substrates to ensure consistency of the thin films deposited on the different substrate materials. Further details are available in Tables S1 and S2.

All characterization, except for XPS and UPS, was conducted on air-exposed thin films. Experimental data was managed and analyzed in a local customized NOMAD Oasis database infrastructure.^45^ Further details on the measurement conditions, instruments and data analysis are available in the SI.

## 3. Computational details

Cu_3_PS_4_ properties were calculated using Density Functional Theory (DFT) using a plane wave basis set as implemented in the VASP^46–50^ software. The exchange–correlation hybrid functional Heyd–Scuseria–Ernzerhof (HSE06)^51,52^ was used in this work, unless otherwise specified. Hybrid functionals significantly alleviate the systematic band gap underestimation problem of semilocal functionals, and generally yield optoelectronic properties in much better agreement with experiment.^53^ All calculations were submitted using the SLURM frontend MyQueue.^54^ The initial structure of Cu_3_PS_4_, extracted from the Materials Project,^55^ entry mp-3934, was relaxed through a geometry optimization. A plane wave energy cut-off of 680 eV was selected for all calculations. All calculations, except that of the effective masses, used a Monkhorst–Pack *k*-point grid of 5 × 5 × 4 Bloch vectors to describe the Brillouin zone. During the structure relaxation, a convergence threshold of 0.01 eV Å^−1^ of force per ion was used.

A non-self-consistent calculation was employed to determine the electronic band structure from the relaxed structure, followed by a frequency-dependent absorption calculation for the absorption spectrum. The sigma broadening factor of 0.02 was used for the band structure calculation, while the absorption spectrum calculation and the orbital-resolved density of states (DOS) calculation were resolved with a sigma broadening factor of 0.1 and plotted with the sumo^56^ package. The conductivity effective masses were calculated at room temperature in the low carrier concentration limit using the BoltzTraP2 ^57^ package on a single point calculation with a (11 × 11 × 11) *k*-point grid.

The charge/bonding properties and thermodynamic stability landscape of the Cu–P–S system (convex hull) were calculated using the Perdew–Burke–Ernzerhof gradient corrected functional for solids (PBESol).^58^ The convex hull was based on in-house calculations on all the known ternary and binary Cu–P–S phases and plotted with the pymatgen package.^59,60^ The charge and bonding analysis was performed using the LOBSTER^61^ package on a single point calculation. LOBSTER extracts local properties by projecting the plane wave basis set onto a local basis set.

## 4. Results

### 4.1 Composition and phase identification

**Fig. 2:**
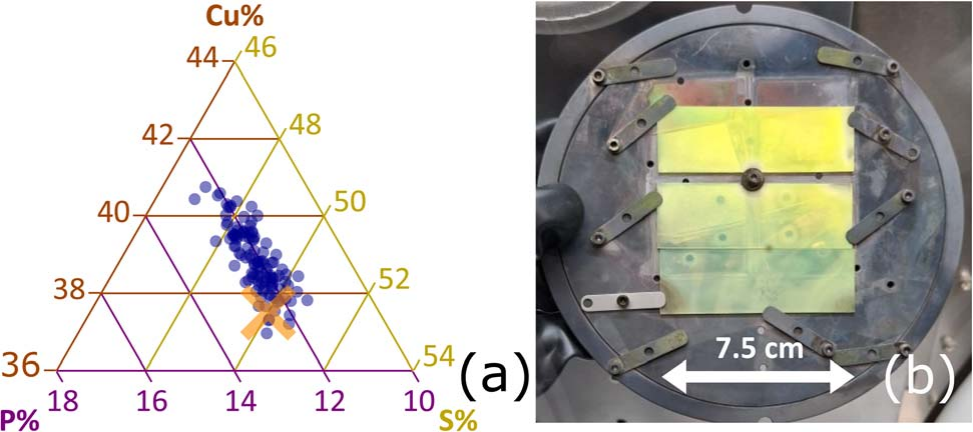
Large-area Cu_3_PS_4_ deposition. (a) Bulk film composition at 120 different points spanning an 8 × 8 cm^2^ area, as measured by EDX. The stoichiometric Cu_3_PS_4_ point is marked with an orange X on top of the data points in the ternary plot. (b) Photograph of a deposited film on the substrate holder.

Depending on the relative fluxes of the precursors, the bulk elemental composition of the Cu–P–S films can either exhibit combinatorial gradients following the geometry of the sources (Fig. 1c–e) or be locked to the Cu_3_PS_4_ composition within the error bar of the EDX measurement (Fig. 2a). At sulfur partial pressures above 0.2 mTorr, the Cu_3_PS_4_ composition is locked over an area of at least 8 × 8 cm^2^ (Fig. 2b).

**Fig. 3:**
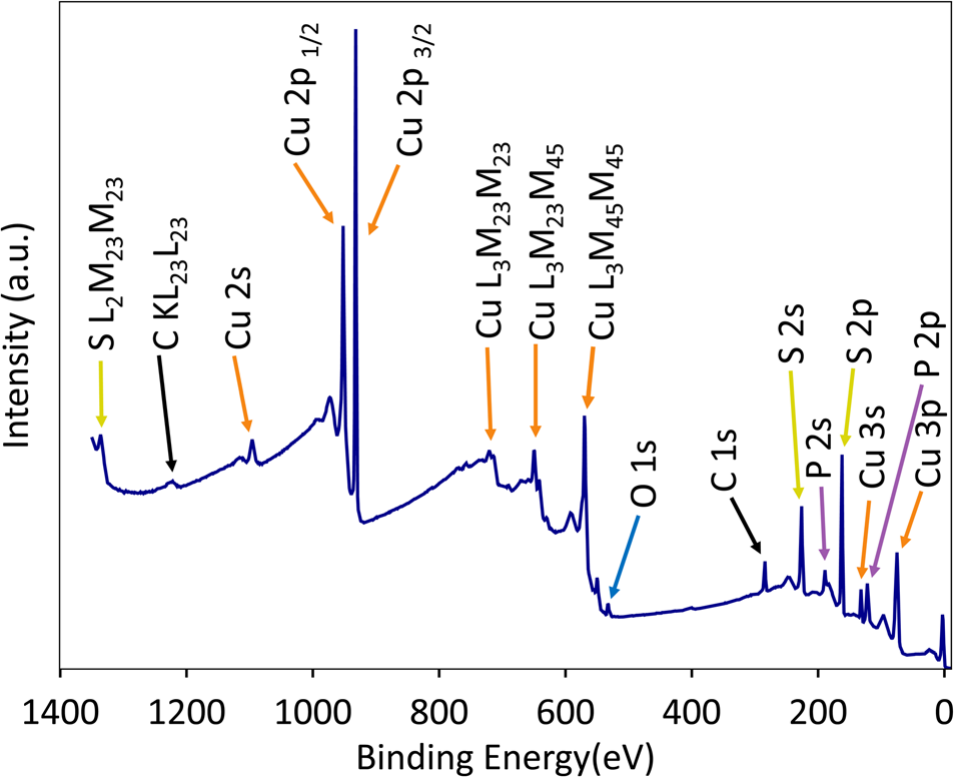
XPS survey scan of an as-deposited enargite Cu_3_PS_4_ film without nominal air exposure. The labeled peaks are either core level or Auger peaks. The remaining features are satellites of such peaks or hybridized valence band states. Different elements are marked with different colors.

For an exemplary film with stoichiometric Cu_3_PS_4_ composition in the bulk, the surface composition is also essentially stoichiometric within the error bar of the XPS measurement (Table 1). Thus, Cu_3_PS_4_ does not exhibit the strongly Cu-poor surfaces typically observed in the related compounds Cu_2_ZnSnS_4_ and CuInS_2_.^62,63^ Oxygen contamination is 2.7% in the bulk after air exposure (EDX, semiquantitative), and 5.1% at the as-deposited surface without nominal exposure to air (XPS). Oxygen is the only impurity conclusively detected by EDX, whereas XPS measurements also reveal adventitious carbon and <0.1% Sb (Table S3) due to parallel work on Sb-containing materials in the same chamber (see SI). Using *in situ* ion-beam sputtering in the XPS chamber, O, C, and Sb appear to be mainly confined to the surface. All other peaks in XPS survey spectra can be indexed to Cu, P, or S core level peaks, Auger peaks, or their satellites (Fig. 3).

**Table 1:** Atomic bulk and surface composition at the same location of a thin film exhibiting the enargite structure. The sum of Cu, P, and S contents is normalized to 100%. The ideal stoichiometric composition is also given

	Cu%	P%	S%
Bulk (EDX)	37.8 ± 0.5	12.1 ± 0.2	50.1 ± 0.7
Surface (XPS)	35.4 ± 1.8	11.9 ± 0.6	52.7 ± 2.6
Stoichiometric	37.5	12.5	50.0

**Fig. 4:**
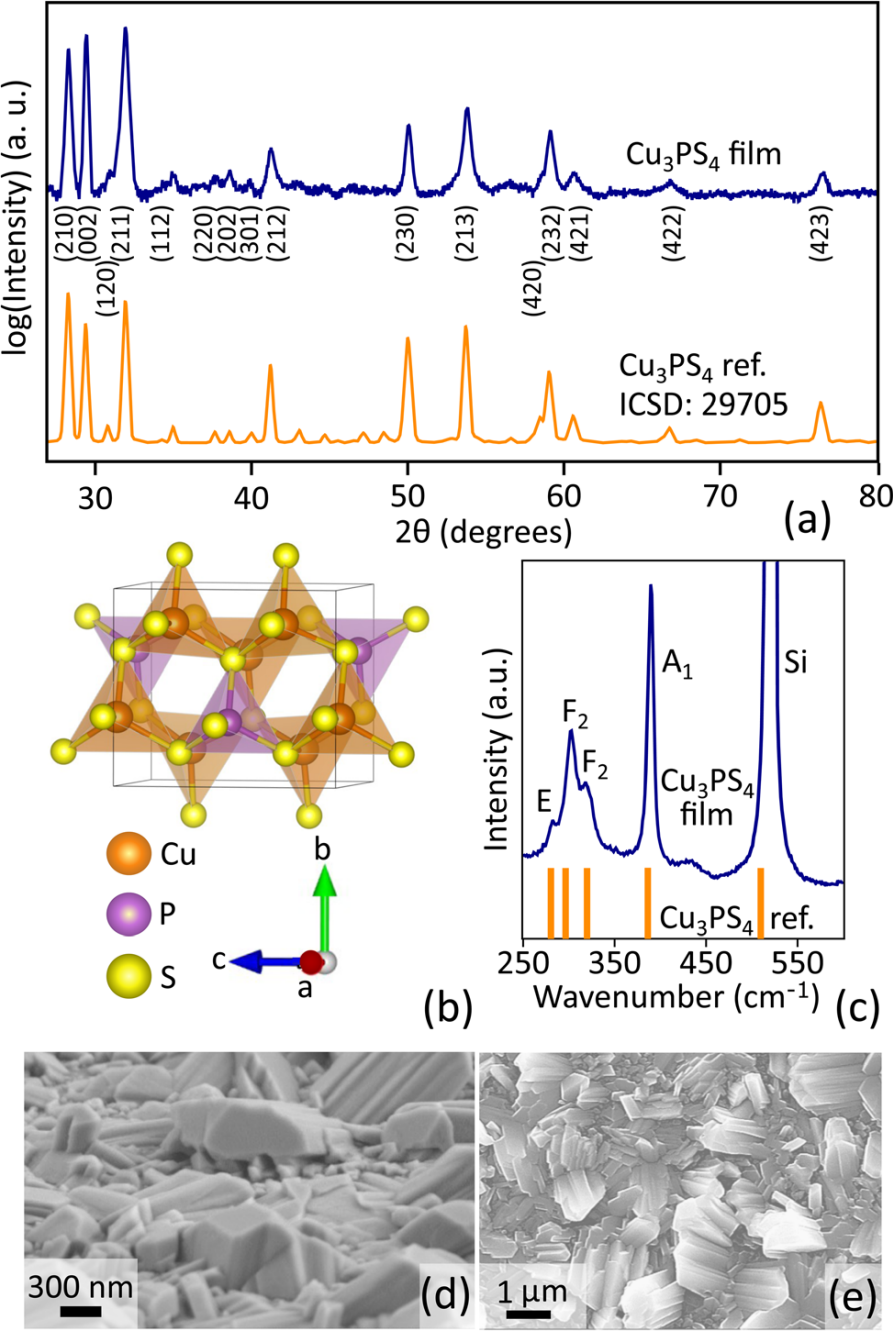
Structural and morphological characterization of Cu_3_PS_4_ films. (a) Experimental XRD pattern of the thin-film sample and simulated pattern from a reference enargite single crystal (ICSD Collection Code: 29705).^38^ (b) Enargite crystal structure of Cu_3_PS_4_ represented as a ball and stick model with polyhedra visualized with VESTA.^64^ (c) Raman spectrum of the thin film and the positions of peaks and mode assignment previously reported for Cu_3_PS_4_.^41,65^ (d) SEM micrograph of the Cu_3_PS_4_ thin film tilted to 20° with respect to the substrate plane. (e) Top view SEM micrograph of the Cu_3_PS_4_ thin film.

Sixteen peaks can be identified with a high degree of confidence in the XRD pattern of a film with stoichiometric Cu_3_PS_4_ composition (Fig. 4a). All these peaks can be indexed to a single-crystal Cu_3_PS_4_ reference (Collection Code: 29705) from the Inorganic Crystal Structure Database (ICSD)^38^ The bulk crystal structure (enargite) is based on the Cu_3_AsS_4_ mineral and belongs to the orthorhombic space group *Pmn*2_1_ (Fig. 4b).^64^ Enargite is a wurtzite-like structure with distorted tetrahedra. The distortions in Cu_3_PS_4_ are rather small and more pronounced in the CuS_4_ tetrahedra, with tetrahedral angles in the 104.4–114.0° range (CuS_4_) and in the 108.3–111.3° range (PS_4_).^38^ It is important to note that P does not act as a P^3−^ anion in Cu_3_PS_4_, in stark contrast with classic phosphide semiconductors like GaP. Instead, P is present as P^5+^ and it does not bond to Cu, but only to S. Enargite Cu_3_PS_4_ can then be seen either as a metal thiophosphate containing the well-known [PS_4_]^3−^ polyanion,^1^ or alternatively as a classic double-cation, single anion sulfide where P^5+^ has a similar role to In^3+^ in CuInS_2_. In fact, bonding and chemical state analysis (shown later in Fig. 8) reveals many parallels between Cu_3_PS_4_ and CuInS_2_.

The relative intensities of the XRD peaks in Fig. 4a are similar to those expected for a randomly oriented Cu_3_PS_4_ powder sample. This indicates a (nearly) random orientation of the grains in the thin film, as also supported by the 2D diffraction image from the XRD measurement (Fig. S2). A LeBail refinement on the XRD pattern (Fig. S3) was performed to extract the unit cell parameters. The lattice parameters (*a* = 7.29, *b* = 6.30, *c* = 6.06) deviate less than 0.5% from the lattice parameters of the reference (*a* = 7.28, *b* = 6.33, *c* = 6.08),^38^ so we conclude that the present film is structurally very similar to a well-defined reference crystal. No additional peaks from impurity phases were observed. The full width at half maximum (FWHM) of the (002) XRD peak in our film is 0.25 °2*θ*, significantly narrower than the XRD peaks in the same °2*θ* region reported for any phosphosulfide thin film demonstrated so far^2,3,9–15^ including nanoparticle-coated Cu_3_PS_4_.^2,3^

The Raman spectrum of one of our enargite Cu_3_PS_4_ films (Fig. 4c) is consistent with previous reports on Cu_3_PS_4_ powders, with well-defined peaks at 282, 299, 318, and 391 cm^−1^ attributed to A_1_, E, and F_2_ modes, expected for a spectroscopically active PS_4_^3−^ tetrahedron with *T*_d_ symmetry.^41,65^ SEM images display a continuous (Fig. S5) polycrystalline thin film with grains of varying sizes and orientations (Fig. 4d and e). This leads to significant surface roughness. The average grain size as inferred from SEM images at different tilts is several hundreds of nm, but the grain size distribution is broad with some grains exceeding 1 μm.

The above discussion is based on single-point measurements, but we systematically observe that any film with Cu_3_PS_4_ stoichiometry within the error bar of the EDX measurement shows enargite XRD peaks without clear secondary phases, regardless of thickness, substrate type, and position in the chamber. Importantly, 120 EDX measurements and 100 XRD measurements done on equally spaced grids across a 8 × 8 cm^2^ deposition area indicate that every point shown in Fig. 2a consists of single-phase enargite Cu_3_PS_4_ within the detection limit of the XRD measurement. Due to the asymmetric geometry of the deposition sources, there is a strong thickness gradient (visible in Fig. 2b), but this gradient can easily be removed by substrate rotation combined with a confocal source geometry.

In general, the proposed thin-film process route involving reactive sputtering of a Cu target under PH_3_ reactive gas and a cracked sulfur beam is able to produce phase-pure Cu_3_PS_4_ films with several desirable features: (i) spatially uniform composition and structure under sufficiently high sulfur partial pressure; (ii) clear potential for large-area coating and fast deposition rates, as the deposition rate from a metallic Cu target can potentially be increased by two orders of magnitude in an industrial setting;^66^ (iii) the largest grain size and narrowest XRD peaks reported so far for a phosphosulfide thin film.^2,3,9–15^ These features are achieved at a deposition temperature (465 °C) lower than the typical growth temperature of chemically related semiconductors CuInS_2_ and Cu_2_ZnSnS_4_ (above 500 °C). In fact, the deposition temperature can be further reduced to 375 °C and still result in a phase-pure enargite Cu_3_PS_4_ film with only a minor effect on XRD peak intensities and widths (Fig. S4). These growth temperatures are compatible with many metals and oxides used as contact and transport layers in optoelectronic and photoelectrochemical devices.

### 4.2 Stability

**Fig. 5:**
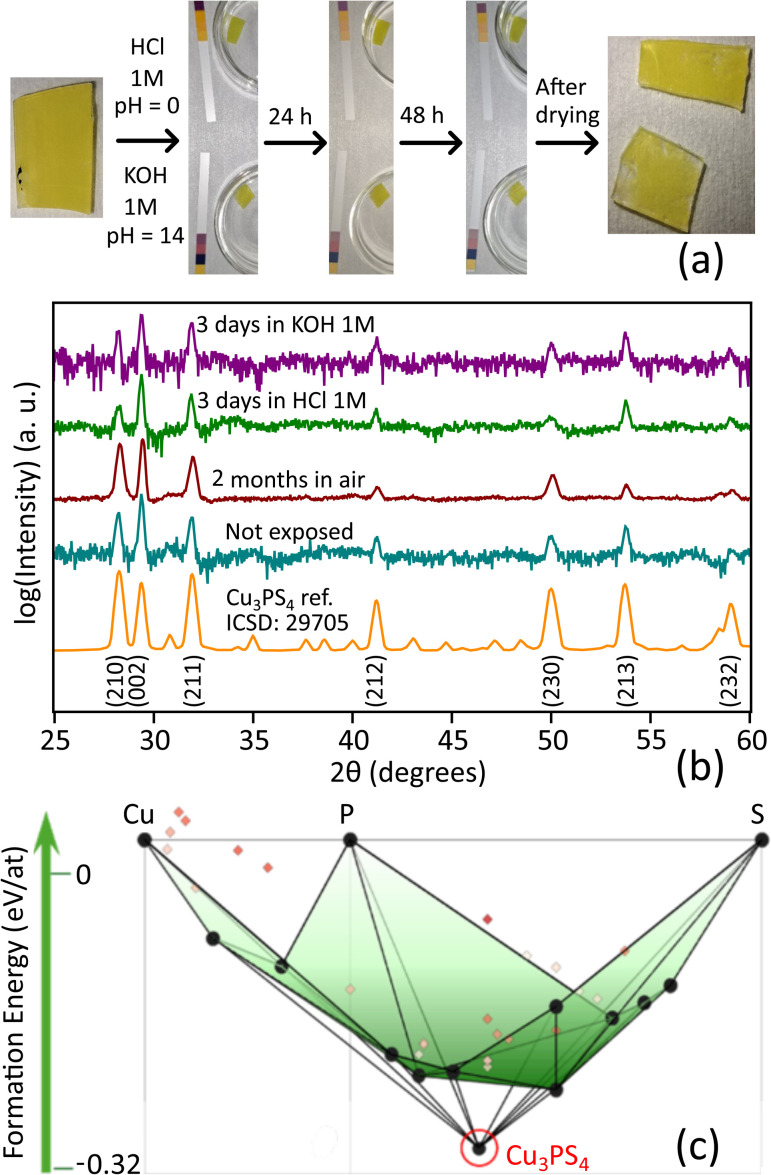
Stability of Cu_3_PS_4_. (a) Photographs of Cu_3_PS_4_ films taken before, during, and after exposure to 1 M HCl and 1 M KOH solutions. (b) XRD patterns of Cu_3_PS_4_ films after prolonged exposure to air, HCl solution with pH = 0, and KOH solution with pH = 14. (c) DFT-calculated convex hull of thermodynamic stability in the Cu–P–S material space. The green surface shows the convex hull in the absence of the Cu_3_PS_4_ phase. This surface is defined by connecting all the thermodynamically stable phases (black dots). Addition of enargite Cu_3_PS_4_ (circled in red) results in substantial energy lowering. The colored markers indicate phases that are not thermodynamically stable.

Cu_3_PS_4_ films are stable in ambient air for at least two months (Fig. 5a). Previous studies reported that bulk Cu_3_PS_4_ powders only dissolved when heated in strong acids or in aqua regia, suggesting high chemical stability.^24^ However, it is unclear from these early investigations if a slow but non-zero etch rate exists for Cu_3_PS_4_. Thin-film samples are an ideal platform to experimentally test slow etching processes. We submerged two thin-film samples on a glass substrate in a 1 M KOH (pH = 14) and 1 M HCl (pH = 0) solution for 72 h. The sample in the HCl solution did not change visibly during 3 days. The sample in the KOH solution looked the same after 24 h. After 72 h, we observed delamination of the film at the edges of the substrate (Fig. 5a). This process was not caused by etching of the Cu_3_PS_4_ film itself, but rather by slow etching of the glass substrate in KOH.^67^ XRD patterns of the HCl and KOH-exposed samples feature the same peaks as a freshly prepared thin-film sample (Fig. 5b), confirming the retention of enargite Cu_3_PS_4_. Since the films are under 200 nm thick, the etch rate of Cu_3_PS_4_ must be negligible or well under 2 nm h^−1^ under highly acidic and highly alkaline conditions, indicating exceptional pH stability at room temperature. Interestingly, a Pourbaix diagram for the Cu–P–S system available on Materials Project predicts Cu_3_PS_4_ to be unstable in aqueous solutions at any pH.^68^ This may indicate that the experimentally observed stability of the material is either due to formation of a surface passivation layer, or due to slow kinetics.

Cu_3_PS_4_ films also possess high irradiation resistance. The films do not show irreversible changes after high doses of sub-band gap laser light (10^4^ sun equivalents at 785 nm for 1 min), above-band gap laser light (3 × 10^3^ sun equivalents at 405 nm for 1 min), high-energy electrons (20 W cm^−2^ power density at 15 keV for 1 min) and X-rays (high-power Cu-Kα radiation focused onto a 500 μm spot for 12 h).

The remarkable stability of Cu_3_PS_4_ may be qualitatively rationalized by inspecting the DFT-calculated Cu–P–S convex hull (Fig. 5c), a three-dimensional surface (green) connecting the thermodynamically stable materials in the Cu–P–S system at 0 K. Cu_3_PS_4_ has a significantly lower formation energy (−0.32 eV per atom) than all other ternaries in the material space. This can be visualized by the large change in the convex hull in the presence *vs.* absence of Cu_3_PS_4_ (Fig. 5c). This well of stability indicates that, once enargite Cu_3_PS_4_ is formed, there is a large energy barrier for bond breakage and decomposition. Although the M_3_PS_4_ composition is present in many other chemical systems, no other metal M presents such a large difference in formation energy between the M_3_PS_4_ composition and the rest of the chemical space (Fig. S8).

### 4.3 Optoelectronic properties

**Fig. 6:**
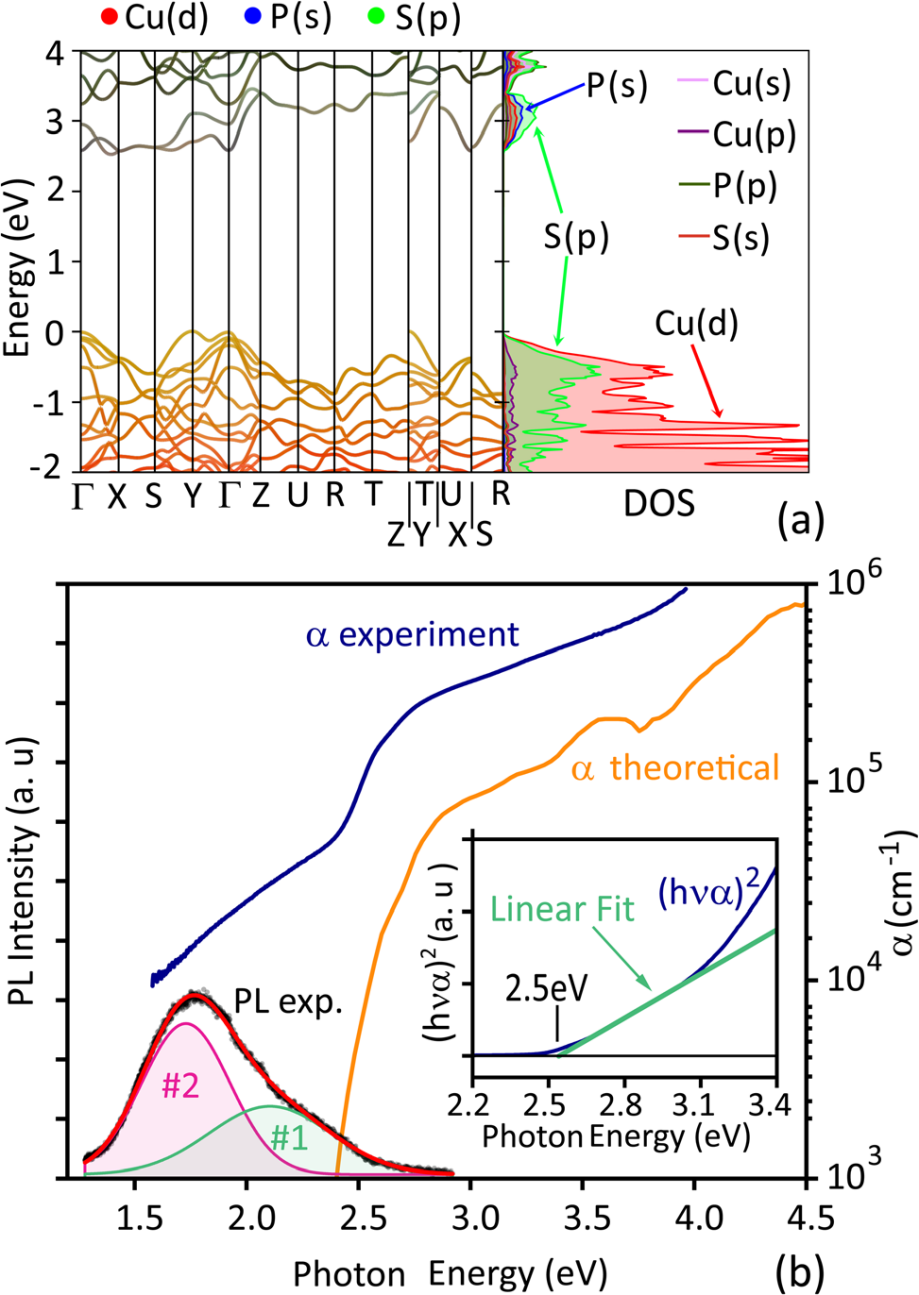
Optoelectronic properties of Cu_3_PS_4_. (a) Left: Band structure calculated at the HSE06 level, colored based on the main contributions from the orbitals of each element. The valence band maximum is situated at Y, while the conduction band minimum is situated close to X. Right: The corresponding orbital-resolved density of states, with the main contributors to the VBM and CBM indicated with arrows. (b) Comparison of the HSE06-calculated absorption coefficient and the experimental absorption coefficient measured on a Cu_3_PS_4_ thin film. The experimental PL spectrum of the film (black) is also shown and is fitted with two Gaussian contributions #1 and #2, whose sum is shown in red. Inset: Tauc plot for direct bandgap semiconductors and corresponding linear fit of the linear segment of the absorption edge, indicating the absorption onset.

The HSE06 band structure calculation of Cu_3_PS_4_ (Fig. 6a) shows it to be an indirect gap semiconductor with a band gap of 2.53 eV, a valence band maximum (VBM) at the Y point of the Brillouin zone and a conduction band minimum (CBM) between the X and the Γ points. However, the energy of the direct gap at the Γ point is only 60 meV higher (2.59 eV). The calculated absorption coefficient (Fig. 6b), which does not include any indirect transitions, reaches 10^5^ cm^−1^ around 0.5 eV above the direct band gap. Thus, above-band gap absorption is quite strong, even compared to common direct gap semiconductors.^69^

In accordance with the calculation, we observe an absorption edge at around 2.5 eV photon energy in the experimental absorption coefficient spectrum α (Fig. 6b). We attribute this edge to the onset of optical transitions across the Cu_3_PS_4_ band gap, consistently with the yellow color of the film (Fig. 2b). We find a broad photon energy (*hν*) range (2.6–3.1 eV) where (*αhν*)^2^ is linear in *hν* (inset of Fig. 6b), in line with the classic behavior expected for a direct-gap semiconductor (Tauc plot). Linear extrapolation of (*αhν*)^2^ to zero yields an estimate of 2.5 eV for the direct gap of Cu_3_PS_4_. With an HSE06-predicted energy difference of only 60 meV between the fundamental indirect gap and the direct gap (Fig. 6a), indirect transitions are very unlikely to be resolved by a Tauc plot for indirect-gap materials. Band gap estimation *via* Tauc plots has little physical basis and can lead to erroneous results,^70,71^ in complex materials exhibiting, *e.g.*, significant band nonparabolicity, anisotropy, and multiple band pockets. To provide an alternative estimate for the experimental band gap, we use the method outlined in the SI to align the computed and experimental absorption coefficients. This procedure yields a direct gap of 2.41 eV and an indirect gap as 2.35 eV. These values are similar to previously reported band gaps (2.36–2.38 eV) for bulk Cu_3_PS_4_ samples.^25,26^

At energies above the main absorption edge, the absorption coefficient swiftly reaches very high values (*e.g.*, 3 × 10^5^ cm^−1^ already at 2.7 eV), surpassing even the highest-efficiency direct band gap photovoltaic semiconductors (GaAs, perovskites, CIGSe).^69^ At energies below the 2.5 eV edge, the measured absorption coefficient remains relatively high (10^4^ cm^−1^ range) even 1 eV below the computationally predicted band gap. It is currently unclear if this sub-band gap absorption is a genuine effect or simply an artifact in the transmission-reflection measurements used to derive *α*. Similar artifacts, even in the relatively high 10^4^ cm^−1^ absorption coefficient range, are well documented in similar materials, their origin being internal light trapping due to surface roughness.^72^ The high roughness of our Cu_3_PS_4_ film (Fig. 4d) is expected to exacerbate this issue.

Photoluminescence (PL) spectroscopy measurements at room temperature reveal two broad Gaussian peaks centered at 1.7 and 2.1 eV with full widths at half maximum (FWHM) around 0.5 eV (Fig. 6b). Thus, the dominant radiative recombination mechanisms in the Cu_3_PS_4_ film involve energy levels well inside the band gap. These transitions could alternatively explain the substantial absorption below the bandgap, and hint that the parasitic absorption may at least partially be a property of the sample, and not a measurement artifact. More detailed studies are needed to resolve this ambiguity.

Effective masses (in units of electron rest mass *m*_0_) are 0.93, 2.68, 0.30 for electrons and 1.24, 1.33, 0.60 for holes, along the *a*, *b*, and *c* axis, respectively. The calculations predict that the *b* axis is the least favorable transport direction in Cu_3_PS_4_, especially for electrons in the conduction band. The direction-averaged effective masses are 1.30*m*_0_ for electrons and 1.06*m*_e_ for holes. These values are higher than in conventional semiconductors (Si, GaAs, CdTe, CuInSe_2_), but lower than in many anisotropic semiconductors such as Sb_2_Se_3_ (ref. 73) for which solar cell devices with photovoltaic (PV) efficiencies above 10% have been reported.

**Fig. 7:**
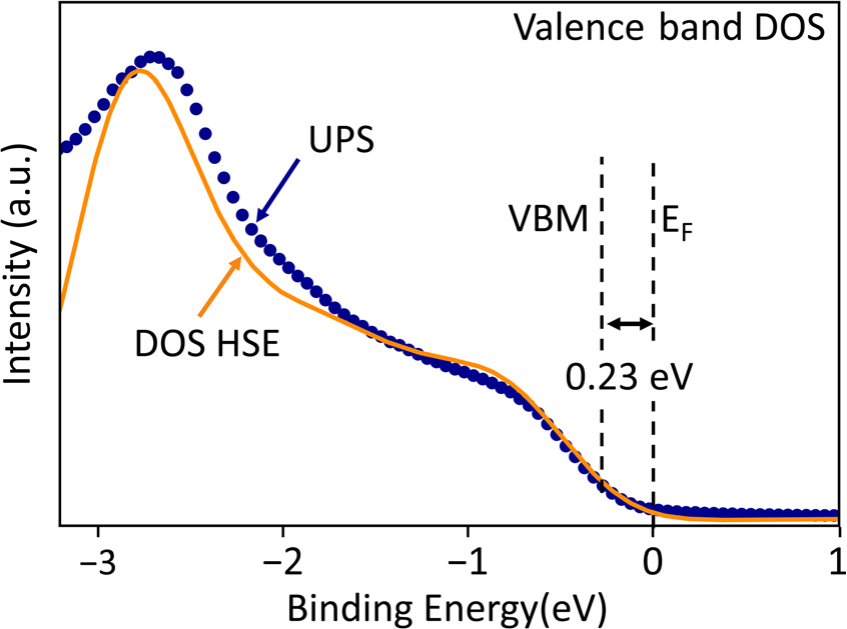
Valence band onset analysis. Measured UPS valence band onset of Cu_3_PS_4_ referenced to the Fermi level *E*_F_ at 0 binding energy. The UPS spectrum is plotted with a broadened DOS from an HS06 calculation on Cu_3_PS_4_. The −0.23 eV shift of the DOS necessary to align it to the UPS spectrum corresponds to the position of the VBM below the Fermi level at the Cu_3_PS_4_ surface, indicating p-type conductivity.

The doping type and carrier concentration at the Cu_3_PS_4_ film surface can be estimated by aligning a UPS valence band spectrum to a DOS calculation (Fig. 7) using the Kraut method^74^ combined with the calculated effective masses of Cu_3_PS_4_. The procedure described in the SI yields a VBM 0.23 eV below the Fermi level, implying p-type conductivity at the Cu_3_PS_4_ surface with a carrier concentration *p* = (6 ± 4) × 10^15^ cm^−3^, in a similar range as the bulk p-type doping densities of 10^16^ − 10^17^ cm^−3^ previously measured on Cu_3_PS_4_ crystals and powders by Hall effect measurements.^25,26^ Typical doping densities of CuInS_2_ and Cu_2_ZnSnS_4_ are in the same range.

Overall, the combination of strong optical absorption, intermediate carrier concentration, and extreme pH stability indicates that Cu_3_PS_4_ may be a suitable candidate for light driven electrochemical reactions in either acid or base. The 2.3–2.5 eV band gap of Cu_3_PS_4_ is typical for various water-splitting photoelectrodes, such as BiVO_4_ and TaON.^75^

### 4.4 Chemical bonding

In this section, we will refer to both oxidation states and charge states of the ions, the distinction being that the former is the formal charge of an ion assuming full charge transfer (ionic limit), and the latter is the actual charge localized on the ion as estimated by experiment or first-principles calculation.^76^ Mulliken charge analysis of Cu_3_PS_4_ predicts average charges of +0.67, +0.55 and −0.64 for Cu, P and S, respectively. This deviation from the formal oxidation states +1, +5, −2 is expected due to the nature of the bonds these elements form. Copper, having a larger electronegativity difference from sulfur than phosphorus, prefers more ionic bonds, donating electrons in the process. Despite its very high formal oxidation state, phosphorus is less ionized than copper due to the similar electronegativity between phosphorus and sulfur. Sulfur, bonded with both copper and phosphorus, falls somewhere in the middle.

These interpretations are confirmed in the integrated Crystal Orbital Bond Index (iCOBI) values calculated with the LOBSTER program. An iCOBI value of 1 corresponds to an ideal covalent single bond, and a value of 0 indicates pure ionic bonding. All Cu–S bonds in Cu_3_PS_4_ have iCOBI values around 0.10 (±0.008), indicating a high degree of ionicity. P–S bonds have values around 0.41 (±0.005), due to a higher degree of covalency in the bond, wherein the overlap of orbitals, and not only electrostatic interactions, contribute to lowering the total energy of the crystal.

**Fig. 8:**
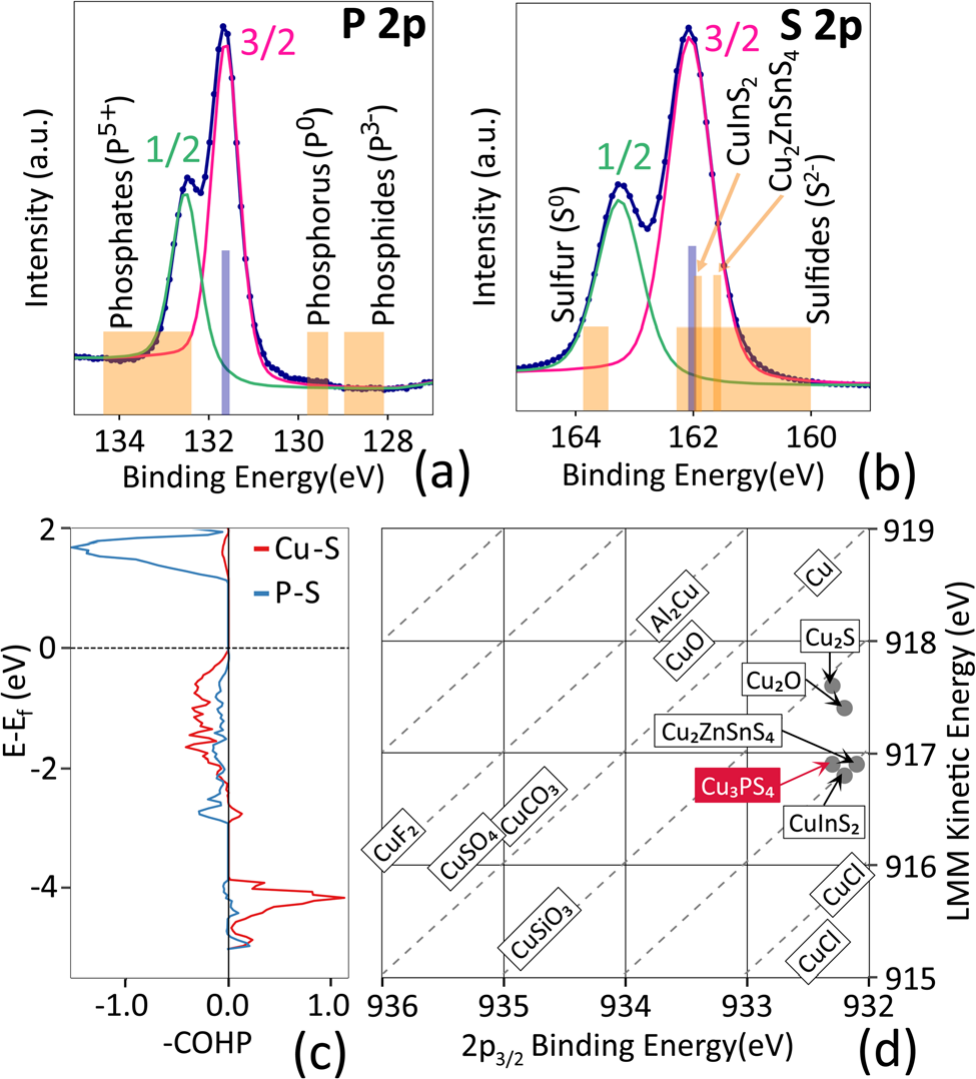
Bonding analysis of Cu_3_PS_4_. (a and b) High-resolution XPS scans (blue line with markers) for the P 2p core level (a) and the S 2p core level (b). Peak fits for the spin–orbit-split 1/2 and 3/2 components are shown in green and pink, respectively. The fitted 2p_3/2_ peak center for Cu_3_PS_4_ is indicated in blue. Literature ranges for various materials according to oxidation states are indicated in orange. (c) Calculated Crystal Orbital Hamilton Population (–COHP) of Cu_3_PS_4_ resolved by type of bond (Cu–S and P–S). (d) Plot of Cu 2p_3/2_ peak position *vs.* Cu LMM Auger peak position (Wagner chemical state plot) for Cu_3_PS_4_ compared to typical literature values.^77^ Values for CuInS_2_ and Cu_2_ZnSnS_4_ are taken from ref. 78 and 79. The sum of the two peak positions is the modified Auger parameter *α*′. The dashed lines are lines of constant *α*′.

The Crystal Orbital Hamilton Population (COHP) from LOBSTER also evaluates the bonding interactions between elements. Negative values of –COHP indicate antibonding interactions, while positive values indicate bonding. The bond-projected COHP across the energies (Fig. 8c) shows that both the VBM and the CBM are dominated by antibonding interactions, which mainly arise from Cu–S bonds at the VBM and from P–S bonds at the CBM. Bonding interactions are only dominant several eV below the VBM.

According to the orbital-projected density of states (Fig. 6a) the specific antibonding interactions at the VBM are between Cu 3d states and S 3p states, as in the better-known Cu sulfide semiconductors CuInS_2_ (chalcopyrite) and Cu_2_ZnSnS_4_ (kesterite).^80–82^ At the CBM, the dominant antibonding interactions are between S 3p states and P 4s states, with a higher P 4s character at the Γ point than at the CBM on the Γ–X path. Again, the situation is analogous to S 3p/In 6s mixing at the CBM of chalcopyrites and S 3p/Sn 6s mixing in kesterites, pointing to the similar role of P, In, and Sn across these copper-based sulfides, and to phosphorus behaving much like a post-transition metal cation.

For an experimental analysis of the elemental charge states and bonding, high-resolution XPS scans of the Cu 2p, P 2p, and S 2p core levels and the Cu LMM Auger peak were performed. Measured profiles, peak fitting, and peak positions are shown in Fig. S7 and Table S3, SI. The P 2p_3/2_ peak center (131.63 eV, Fig. 8a) lies between elemental P (oxidation state of zero) and phosphates (same +5 oxidation state as in thiophosphates).^77^ A more covalent bond to S compared to O results in a lower positive charge on P and can explain the shift to lower binding energy. The S 2p_3/2_ peak position (162.06 eV, Fig. 8b) is on the high binding energy side for sulfide compounds (sulfur in −2 oxidation state^77^), indicating a less negative charge. This suggests that sulfur is more covalently bonded in Cu_3_PS_4_ than in most metal sulfides, in accordance with the relatively high degree of covalency identified for the P–S bond through iCOBI analysis.

Chemical state analysis of Cu is aided by visualizing the Cu 2p_3/2_ binding energy against the Cu LMM Auger kinetic energy in a Wagner plot (Fig. 8d).^83,84^ The Cu 2p_3/2_ peak position is consistent with the presence of Cu in the (diamagnetic) +1 oxidation state, rather than the (paramagnetic) +2 oxidation state. This is also confirmed by the absence of satellite peaks in the Cu 2p region, a hallmark of paramagnetic Cu^2+^.^77^ When examining the Cu LMM Auger peak position in different Cu^1+^ compounds, the ordering Cu–Cu_2_S–Cu_2_O–Cu_3_PS_4_–CuCl, may clash with chemical intuition, since it indicates that Cu is more highly charged in Cu_3_PS_4_ than in Cu_2_O, despite oxygen being more electronegative than sulfur. This trend can be explained by the inductive effect,^85^ wherein the least electronegative species in a ternary compound (here, copper) donates additional charge to enhance covalency between the two other elements (here, phosphorus and sulfur). The position of Cu states in a Wagner plot is very similar in Cu_3_PS_4_, CuInS_2_ and Cu_2_ZnSnS_4_ (Fig. 8d), indicating a similar inductive effect in all these compounds. We conclude that the combination of XPS measurements and computational tools for real-space analysis provide us with a consistent picture of bonding interactions and charge states in Cu_3_PS_4._ Cu_3_PS_4_ has remarkable chemical similarity to other compounds (CuInS_2_ and Cu_2_ZnSnS_4_) with high demonstrated PV efficiencies.

## 5. Conclusion

We showed that the proposed DADMARS method can overcome the typical challenges in the growth of phosphorus- and sulfur-containing thin films that have so far hindered the exploration of this fascinating group of materials. Leveraging DADMARS, we deposited thin films within the earth-abundant Cu–P–S material system, both as compositionally graded samples for high-throughput materials research and as large-area Cu_3_PS_4_ films for device applications. Cu_3_PS_4_ films were continuous, phase-pure within the limits of XRD, with low oxygen contamination levels, and had larger crystal grains than any previously deposited phosphosulfide film known to us. Since DADMARS relies on widely available metallic sputter targets, we argue that it may be a viable synthesis route for the deposition of virtually any phosphosulfide thin film in the future.

Cu_3_PS_4_ films showed many similarities to well-known Cu-based multinary sulfide semiconductors such as CuInS_2_ and Cu_2_ZnSnS_4_. All these materials feature a classic corner-sharing tetrahedral backbone, similar frontier orbital interactions, an antibonding character for both their valence band and conduction band, very similar XPS-determined charge states, and moderate p-type conductivity. The feature that sets Cu_3_PS_4_ apart from most semiconductors is that a moderate deposition temperature is sufficient to form large-grained films, while at the same time the films show remarkable stability in air, under irradiation, and under a very broad pH range (from pH 0 to pH 14).

With a band gap in the 2.3–2.5 eV range, strong light absorption, and measurable room temperature photoluminescence, Cu_3_PS_4_ emerges as a compelling candidate material for solar fuels and solar-driven electrochemical reactions under a very wide range of pH conditions. To potentially improve the optoelectronic properties, a two-step process consisting of low-temperature deposition of amorphous Cu_3_PS_4_ films followed by annealing may prove beneficial.

## Author contributions

L. A. M.: data curation and investigation (experimental), conceptualization, writing. J. S. R.: data curation and investigation (computational). E. B.: investigation (absorption coefficient, PL). G. D.: formal analysis (LeBail refinement). J. G.: resources (Raman). I. E. C.: supervision, resources, software. A. C.: conceptualization, methodology, writing, supervision.

## Conflicts of interest

There are no conflicts to declare.

## Supplementary Material

SC-OLF-D5SC05882A-s001

## Data Availability

Data for this article including all presented measurements and code to generate plots are available on NOMAD. DOI: https://nomad-lab.eu/prod/v1/gui/user/uploads/upload/id/wzLDK8MCSTevIUsqWA5CTA. Supplementary information is available. See DOI: https://doi.org/10.1039/d5sc05882a.

## References

[cit1] Mittmann L. A., Crovetto A. (2024). Phosphosulfide semiconductors for optoelectronics and solar energy conversion. J. Phys. Mater..

[cit2] Yin X., McClary S. A., Song Z., Zhao D., Graeser B., Wang C., Shrestha N., Wang X., Chen C., Li C., Subedi K. K., Ellingson R. J., Tang W., Agrawal R., Yan Y. (2019). A Cu 3 PS 4 nanoparticle hole selective layer for efficient inverted perovskite solar cells. J. Mater. Chem. A.

[cit3] Sheets E. J., Yang W.-C., Balow R. B., Wang Y., Walker B. C., Stach E. A., Agrawal R. (2015). An in situ phosphorus source for the synthesis of Cu 3 P and the subsequent conversion to Cu 3 PS 4 nanoparticle clusters. J. Mater. Res..

[cit4] Tiwari D., Alibhai D., Cherns D., Fermin D. J. (2020). Crystal and Electronic Structure of Bismuth Thiophosphate, BiPS 4 : An Earth-Abundant Solar Absorber. Chem. Mater..

[cit5] Liu H., He P., Wang G., Liang Y., Wang C., Fan L.-Z. (2022). Thin, flexible sulfide-based electrolyte film and its interface engineering for high performance solid-state lithium metal batteries. Chem. Eng. J..

[cit6] Kang S., Kim K., Kim B. H., Kim J., Sim K. I., Lee J.-U., Lee S., Park K., Yun S., Kim T., Nag A., Walters A., Garcia-Fernandez M., Li J., Chapon L., Zhou K.-J., Son Y.-W., Kim J. H., Cheong H., Park J.-G. (2020). Coherent many-body exciton in van der Waals antiferromagnet NiPS3. Nature.

[cit7] Yang Y., Liu J., Zhao C., Liang Q., Dong W., Shi J., Wang P., Kong D., Lv L., Jia L., Wang D., Huang C., Zheng S., Wang M., Liu F., Yu P., Qiao J., Ji W., Zhou J. (2024). A Universal Strategy for Synthesis of 2D Ternary Transition Metal Phosphorous Chalcogenides. Adv. Mater..

[cit8] Zhou J., Zhu C., Zhou Y., Dong J., Li P., Zhang Z., Wang Z., Lin Y.-C., Shi J., Zhang R., Zheng Y., Yu H., Tang B., Liu F., Wang L., Liu L., Liu G.-B., Hu W., Gao Y., Yang H., Gao W., Lu L., Wang Y., Suenaga K., Liu G., Ding F., Yao Y., Liu Z. (2022). Composition and phase engineering of metal chalcogenides and phosphorous chalcogenides. Nat. Mater..

[cit9] Shaddad M. N., Arunachalam P., Hezam M., BinSaeedan N. M., Gimenez S., Bisquert J., Al-Mayouf A. M. (2023). Facile fabrication of heterostructured BiPS4-Bi2S3-BiVO4 photoanode for enhanced stability and photoelectrochemical water splitting performance. J. Catal..

[cit10] Bogomolov A. A., Solnyshkin A. V., Kiselev D. A., Raevskii I. P., Shonov V. Y., Sandzhiev D. N. (2008). Temperature behavior of the photovoltaic and pyroelectric responses of Sn2P2S6 semiconductor ferroelectric films. J. Surf. Invest.: X-ray, Synchrotron. Neutron Tech..

[cit11] Quan Z., Hirayama M., Sato D., Zheng Y., Yano T., Hara K., Suzuki K., Hara M., Kanno R. (2017). Effect of excess Li 2 S on electrochemical properties of amorphous li 3 ps 4 films synthesized by pulsed laser deposition. J. Am. Ceram. Soc..

[cit12] Ito Y., Konishi M., Noi K., Deguchi M., Hayashi A., Tatsumisago M. (2018). Sodium thiophosphate electrolyte thin films prepared by pulsed laser deposition for bulk-type all-solid-state sodium rechargeable batteries. J. Ceram. Soc. Jpn..

[cit13] Ito Y., Sakuda A., Ohtomo T., Hayashi A., Tatsumisago M. (2014). Li4GeS4–Li3PS4 electrolyte thin films with highly ion-conductive crystals prepared by pulsed laser deposition. J. Ceram. Soc. Jpn..

[cit14] Sultana U. K., O'Mullane A. P. (2018). Electrochemical Formation of Amorphous Molybdenum Phosphosulfide for Enabling the Hydrogen Evolution Reaction in Alkaline and Acidic Media. ACS Appl. Energy Mater..

[cit15] Musavigharavi P., Kurnia F., Xie L., Park C. K. Y., Ng Y. H., He J., Hart J. N., Valanoor N. (2021). ZnS-GaP Solid Solution Thin Films with Enhanced Visible-Light Photocurrent. ACS Appl. Energy Mater..

[cit16] Baranowski L. L., Zawadzki P., Christensen S., Nordlund D., Lany S., Tamboli A. C., Gedvilas L., Ginley D. S., Tumas W., Toberer E. S., Zakutayev A. (2014). Control of Doping in Cu2SnS3 through Defects and Alloying. Chem. Mater..

[cit17] Adeleye D., Sood M., Valluvar Oli A., Törndahl T., Hultqvist A., Vanderhaegen A., Lanzoni E. M., Hu Y., Kusch G., Melchiorre M., Redinger A., Oliver R. A., Siebentritt S. (2025). Wide-Bandgap Cu(In, Ga)S2 Solar Cell: Mitigation of Composition Segregation in High Ga Films for Better Efficiency. Small.

[cit18] Shin B., Gunawan O., Zhu Y., Bojarczuk N. A., Chey S. J., Guha S. (2013). Thin film solar cell with 8.4% power conversion efficiency using an earth-abundant Cu 2 ZnSnS 4 absorber. Prog. Photovolt.: Res. Appl..

[cit19] Crovetto A., Kojda D., Yi F., Heinselman K. N., LaVan D. A., Habicht K., Unold T., Zakutayev A. (2022). Crystallize It before It Diffuses: Kinetic Stabilization of Thin-Film Phosphorus-Rich Semiconductor CuP2. J. Am. Chem. Soc..

[cit20] Schnepf R. R., Crovetto A., Gorai P., Park A., Holtz M., Heinselman K. N., Bauers S. R., Tellekamp M. B., Zakutayev A., Greenaway A. L., Toberer E. S., Tamboli A. C. (2022). Reactive phosphine combinatorial co-sputtering of cation disordered ZnGeP2 films. J. Mater. Chem. C.

[cit21] Willis J., Bravić I., Schnepf R. R., Heinselman K. N., Monserrat B., Unold T., Zakutayev A., Scanlon D. O., Crovetto A. (2022). Prediction and realisation of high mobility and degenerate p-type conductivity in CaCuP thin films. Chem. Sci..

[cit22] Crovetto A., Adamczyk J. M., Schnepf R. R., Perkins C. L., Hempel H., Bauers S. R., Toberer E. S., Tamboli A. C., Unold T., Zakutayev A. (2022). Boron Phosphide Films by Reactive Sputtering: Searching for a P-Type Transparent Conductor. Adv. Mater. Interfaces.

[cit23] Crovetto A., Unold T., Zakutayev A. (2023). Is Cu3–xP a Semiconductor, a Metal, or a
Semimetal?. Chem. Mater..

[cit24] Glatzel E. (1893). Über normale Sulfophosphate. Z. Anorg. Chem..

[cit25] Marzik J. V., Hsieh A. K., Dwight K., Wold A. (1983). Photoelectronic properties of Cu3PS4 and Cu3PS3Se single crystals. J. Solid State Chem..

[cit26] Itthibenchapong V., Kokenyesi R. S., Ritenour A. J., Zakharov L. N., Boettcher S. W., Wager J. F., Keszler D. A. (2013). Earth-abundant Cu-based chalcogenide semiconductors as photovoltaic absorbers. J. Mater. Chem. C.

[cit27] Shi T., Yin W.-J., Al-Jassim M., Yan Y. (2013). Structural, electronic, and optical properties of Cu 3 -V-VI 4 compound semiconductors. Appl. Phys. Lett..

[cit28] Pauporté Th., Lincot D. (1995). Electrical, optical and photoelectrochemical properties of natural enargite, Cu3AsS4. Adv. Mater. Opt. Electron..

[cit29] McClary S. A., Andler J., Handwerker C. A., Agrawal R. (2017). Solution-processed copper arsenic sulfide thin films for photovoltaic applications. J. Mater. Chem. C.

[cit30] McClary S. A., Taheri M. M., Blach D. D., Pradhan A. A., Li S., Huang L., Baxter J. B., Agrawal R. (2020). Nanosecond carrier lifetimes in solution-processed enargite (Cu 3 AsS 4) thin films. Appl. Phys. Lett..

[cit31] Tripathy D., Sampath S. (2020). Understanding the high capacity contributions of Cu3PS4 towards lithium storage. J. Power Sources.

[cit32] Zhang Z., Mazzio K. A., Riegger L. M., Brehm W., Janek J., Sann J., Adelhelm P. (2023). Copper Thiophosphate (Cu3PS4) as an Electrode Material for Lithium Solid-State Batteries with Lithium Thiophosphate (β–Li3PS4) Electrolyte. Energy Technol..

[cit33] Tripathy D., Sampath S. (2023). Electrochemical performance of copper phosphosulfide (Cu3PS4) towards magnesium ion storage. Electrochim. Acta.

[cit34] Brehm W., Santhosha A. L., Zhang Z., Neumann C., Turchanin A., Martin A., Pinna N., Seyring M., Rettenmayr M., Buchheim J. R., Adelhelm P. (2020). Copper Thiophosphate (Cu3PS4) as Electrode for Sodium-Ion Batteries with Ether Electrolyte. Adv. Funct. Mater..

[cit35] Ho S.-F., Tuan H.-Y. (2023). Cu3PS4: a sulfur-rich metal phosphosulfide with superior ionic diffusion channel for high-performance potassium ion batteries/hybrid capacitors. Chem. Eng. J..

[cit36] Coleman N., Liyanage I. A., Lovander M. D., Leddy J., Gillan E. G. (2022). Facile Solvent-Free Synthesis of Metal Thiophosphates and Their Examination as Hydrogen Evolution Electrocatalysts. Molecules.

[cit37] Cho M., Ju H., Bae S., Bong S., Lee J. (2024). Scalable ammonia synthesis on the modified crystal structure of Cu3PS4 electrocatalyst. Electrochim. Acta.

[cit38] Yang Y., Zhang B., Wu X., Wu K. (2021). A series of M3PS4 (M = Ag, Cu and Ag/Cu) thiophosphates with diamond-like structures exhibiting large second harmonic generation responses and moderate ion conductivities. Dalton Trans..

[cit39] Song B.-J., Ma Z., Li B., Wu X.-T., Lin H., Zhu Q.-L. (2021). Structural Modulation from Cu3PS4 to Cu5Zn0.5P2S8: Single-Site Aliovalent-Substitution-Driven Second-Harmonic-Generation Enhancement. Inorg. Chem..

[cit40] Tripathy D., Kumar R., Pareek P., Sampath S. (2024). A Copper Phosphosulfide-Based Highly Sensitive Ammonia Gas Sensor at Room Temperature. IEEE Sens. J..

[cit41] Pätzmann U., Brockner W. (1983). Schwingungsspektren von Ag3PS4 und Cu3PS4/Vibrational Spectra of Ag3PS4 and Cu3PS4. Z. Naturforsch., A.

[cit42] Nitsche R., Wild P. (1970). Crystal growth of metal-phosphorus-sulfur compounds by vapor transport. Mater. Res. Bull..

[cit43] Jackson A. J., Tiana D., Walsh A. (2016). A universal chemical potential for sulfur vapours. Chem. Sci..

[cit44] Hoye R. L. Z., Schulz P., Schelhas L. T., Holder A. M., Stone K. H., Perkins J. D., Vigil-Fowler D., Siol S., Scanlon D. O., Zakutayev A., Walsh A., Smith I. C., Melot B. C., Kurchin R. C., Wang Y., Shi J., Marques F. C., Berry J. J., Tumas W., Lany S., Stevanović V., Toney M. F., Buonassisi T. (2017). Perovskite-Inspired Photovoltaic Materials: Toward Best Practices in Materials Characterization and Calculations. Chem. Mater..

[cit45] Scheidgen M., Himanen L., Ladines A. N., Sikter D., Nakhaee M., Fekete Á., Chang T., Golparvar A., Márquez J. A., Brockhauser S., Brückner S., Ghiringhelli L. M., Dietrich F., Lehmberg D., Denell T., Albino A., Näsström H., Shabih S., Dobener F., Kühbach M., Mozumder R., Rudzinski J. F., Daelman N., Pizarro J. M., Kuban M., Salazar C., Ondračka P., Bungartz H.-J., Draxl C. (2023). NOMAD: A distributed web-based platform for managing materials science research data. J. Open Source Softw..

[cit46] Kresse G., Hafner J. (1993). Ab initio molecular dynamics for liquid metals. Phys. Rev. B: Condens. Matter Mater. Phys..

[cit47] Kresse G., Furthmüller J. (1996). Efficient iterative schemes for ab initio total-energy calculations using a plane-wave basis set. Phys. Rev. B: Condens. Matter Mater. Phys..

[cit48] Kresse G., Furthmüller J. (1996). Efficiency of ab-initio total energy calculations for metals and semiconductors using a plane-wave basis set. Comput. Mater. Sci..

[cit49] Kresse G., Hafner J. (1994). Ab initio molecular-dynamics simulation of the liquid-metal-amorphous-semiconductor transition in germanium. Phys. Rev. B: Condens. Matter Mater. Phys..

[cit50] Gajdoš M., Hummer K., Kresse G., Furthmüller J., Bechstedt F. (2006). Linear optical properties in the projector-augmented wave methodology. Phys. Rev. B: Condens. Matter Mater. Phys..

[cit51] Krukau A. V., Vydrov O. A., Izmaylov A. F., Scuseria G. E. (2006). Influence of the exchange screening parameter on the performance of screened hybrid functionals. J. Chem. Phys..

[cit52] Heyd J., Scuseria G. E., Ernzerhof M. (2003). Hybrid functionals based on a screened Coulomb potential. J. Chem. Phys..

[cit53] Borlido P., Schmidt J., Huran A. W., Tran F., Marques M. A. L., Botti S. (2020). Exchange-correlation functionals for band gaps of solids: benchmark, reparametrization and machine learning. npj Comput. Mater..

[cit54] Mortensen J. J., Gjerding M., Thygesen K. S. (2020). MyQueue: Task and workflow scheduling system. J. Open Source Softw..

[cit55] Jain A., Ong S. P., Hautier G., Chen W., Richards W. D., Dacek S., Cholia S., Gunter D., Skinner D., Ceder G., Persson K. A. (2013). Commentary: The Materials Project: A materials genome approach to accelerating materials innovation. APL Mater..

[cit56] Ganose A. M., Jackson A. J., Scanlon D. O. (2018). sumo: Command-line tools for plotting and analysis of periodic *ab initio* calculations. J. Open Source Softw..

[cit57] Madsen G. K. H., Carrete J., Verstraete M. J. (2018). BoltzTraP2, a program for interpolating band structures and calculating semi-classical transport coefficients. Comput. Phys. Commun..

[cit58] Perdew J. P., Ruzsinszky A., Csonka G. I., Vydrov O. A., Scuseria G. E., Constantin L. A., Zhou X., Burke K. (2008). Restoring the Density-Gradient Expansion for Exchange in Solids and Surfaces. Phys. Rev. Lett..

[cit59] Ong S. P., Richards W. D., Jain A., Hautier G., Kocher M., Cholia S., Gunter D., Chevrier V. L., Persson K. A., Ceder G. (2013). Python Materials Genomics (pymatgen): A robust, open-source python library for materials analysis. Comput. Mater. Sci..

[cit60] Ong S. P., Jain A., Hautier G., Kang B., Ceder G. (2010). Thermal stabilities of delithiated olivine MPO4 (M = Fe, Mn) cathodes investigated using first principles calculations. Electrochem. Commun..

[cit61] Maintz S., Deringer V. L., Tchougréeff A. L., Dronskowski R. (2016). LOBSTER: A tool to extract chemical bonding from plane-wave based DFT. J. Comput. Chem..

[cit62] Bär M., Schubert B.-A., Marsen B., Krause S., Pookpanratana S., Unold T., Weinhardt L., Heske C., Schock H.-W. (2011). Native oxidation and Cu-poor surface structure of thin film Cu 2 ZnSnS 4 solar cell absorbers. Appl. Phys. Lett..

[cit63] Scheer R., Lewerenz H. -J. (1995). Formation of secondary phases in evaporated CuInS2 thin films: A surface analytical study. J. Vac. Sci. Technol., A.

[cit64] Momma K., Izumi F. (2011). VESTA 3 for three-dimensional visualization of crystal, volumetric and morphology data. J. Appl. Crystallogr..

[cit65] Sala O., Temperini M. L. A. (1975). Resonance raman effect of solid copper thiophosphate. Chem. Phys. Lett..

[cit66] Wiatrowski A., Posadowski W. M., Radzimski Z. J. (2008). Pulsed-DC selfsputtering of copper. J. Phys.: Conf. Ser..

[cit67] Walczak W., Smith N., Venkatachalam S. (2021). P-36: Effective Cleaning of Glass Substrates. SID Symp. Dig. Tech. Pap..

[cit68] Patel A. M., Nørskov J. K., Persson K. A., Montoya J. H. (2019). Efficient Pourbaix diagrams of many-element compounds. Phys. Chem. Chem. Phys..

[cit69] Nishiwaki M., Nagaya K., Kato M., Fujimoto S., Tampo H., Miyadera T., Chikamatsu M., Shibata H., Fujiwara H. (2018). Tail state formation in solar cell materials: First principles analyses of zincblende, chalcopyrite, kesterite, and hybrid perovskite crystals. Phys. Rev. Mater..

[cit70] Dolgonos A., Mason T. O., Poeppelmeier K. R. (2016). Direct optical band gap measurement in polycrystalline semiconductors: A critical look at the Tauc method. J. Solid State Chem..

[cit71] Kavanagh S. R., Savory C. N., Scanlon D. O., Walsh A. (2021). Hidden spontaneous polarisation in the chalcohalide photovoltaic absorber Sn2SbS2I3. Mater. Horiz..

[cit72] Rey G., Spindler C., Babbe F., Rachad W., Siebentritt S., Nuys M., Carius R., Li S., Platzer-Björkman C. (2018). Absorption Coefficient of a Semiconductor Thin Film from Photoluminescence. Phys. Rev. Appl..

[cit73] Wang X., Li Z., Kavanagh S. R., Ganose A. M., Walsh A. (2022). Lone pair driven anisotropy in antimony chalcogenide semiconductors. Phys. Chem. Chem. Phys..

[cit74] Kraut E. A., Grant R. W., Waldrop J. R., Kowalczyk S. P. (1980). Precise Determination of the Valence-Band Edge in X-Ray Photoemission Spectra: Application to Measurement of Semiconductor Interface Potentials. Phys. Rev. Lett..

[cit75] Sivula K., van de Krol R. (2016). Semiconducting materials for photoelectrochemical energy conversion. Nat. Rev. Mater..

[cit76] Walsh A., Sokol A. A., Buckeridge J., Scanlon D. O., Catlow C. R. A. (2018). Oxidation states and ionicity. Nat. Mater..

[cit77] Moulder J. F. , Stickle W. F., Sobol P. E., Bomben K. D., Chastain J., King Jr R. C. and Physical Electronics, Incorporation, Handbook of X-Ray Photoelectron Spectroscopy: A Reference Book of Standard Spectra for Identification and Interpretation of XPS Data, Physical Electronics, Eden Prairie, Minn., 1995

[cit78] Scheer R., Lewerenz H. J. (1994). Photoemission study of evaporated CuInS2 thin films. II. Electronic surface structure. J. Vac. Sci. Technol., A.

[cit79] Köhler L. , Doctoral thesis, BTU Cottbus, Senftenberg, 2017

[cit80] Persson C. (2010). Electronic and optical properties of Cu[sub 2]ZnSnS[sub 4] and Cu[sub 2]ZnSnSe[sub 4]. J. Appl. Phys..

[cit81] Zhang S. B., Wei S.-H., Zunger A., Katayama-Yoshida H. (1998). Defect physics of the CuInSe2 chalcopyrite semiconductor. Phys. Rev. B: Condens. Matter Mater. Phys..

[cit82] Siebentritt S., Igalson M., Persson C., Lany S. (2010). The electronic structure of chalcopyrites—bands, point defects and grain boundaries. Prog. Photovolt.: Res. Appl..

[cit83] Wagner C. D. (1975). Chemical shifts of Auger lines, and the Auger parameter. Faraday Discuss. Chem. Soc..

[cit84] Biesinger M. C. (2017). Advanced analysis of copper X-ray photoelectron spectra. Surf. Interface Anal..

[cit85] Etourneau J., Portier J., Ménil F. (1992). The role of the inductive effect in solid state chemistry: how the chemist can use it to modify both the structural and the physical properties of the materials. J. Alloys Compd..

