# Supplementary material for: Large-area thin-film synthesis of photoactive Cu3PS4 thiophosphate semiconductor with 0–14 pH stability range
Source: Chem Sci. 2025 Oct 14;16(46):21862–73. doi: 10.1039/d5sc05882a (PMC12536356; doi:10.1039/d5sc05882a)
Supplement: SC-016-D5SC05882A-s001 [file SC-016-D5SC05882A-s001.pdf]

Supplementary Information (SI) for Chemical Science.
This journal is © The Royal Society of Chemistry 2025

Supplementary information for Large-area thin-film synthesis of photoactive Cu_3PS_4 thiophosphate semiconductor with 0-14 pH stability range

Lena A. Mittmann*[1], Javier Sanz Rodrigo[1], Eugène Bertin[1], Giulia Dalmonte[2], Jean-Claude Grivel[2], Ivano E. Castelli[2] and Andrea Crovetto*[1]

[1] National Centre for Nano Fabrication and Characterization (DTU Nanolab), Technical University of Denmark, 2800 Kongens Lyngby, Denmark
[2] Department of Energy Conversion and Storage (DTU Energy), Technical University of Denmark, 2800 Kongens Lyngby, Denmark

E-mail: mittma@dtu.dk, ancro@dtu.dk

Extended experimental details

Equipment: We set up the deposition chamber so that the targets point to one side of the substrate to maximize the composition gradients of the films while covering the full substrate area. Cu was deposited from an unbalanced magnetron sputter source. The deposition geometry highlighting the target to substrate distances as well as the angles of the directional targets are displayed in detail in Figure S1. An S-cracker was used to achieve a high chemical potential of sulfur during deposition. The main components of the S-cracker are the effusion cell (Zone 1), a quartz ball valve with a user-controlled duty cycle (Zone 2) and a cracking zone (Zone 3). In the effusion cell the solid sulfur pellets are heated until an equilibrium vapor pressure is reached. The pulsed valve controls the sulfur vapor flux at the substrate by adjustments to the duty cycle. In practice, the two adjustable parameters of this valve are the valve "on" time per pulse and the pulsing frequency. The pulsing frequency determines how often the valve opens per unit time, while the valve "on" time determines how long the valve stays open each time. The duty cycle is then equal to the valve "on" time divided by the pulsing frequency. The sulfur vapor passes through the cracking zone after the valve where the temperature is increased to crack the S_8 rings into smaller more reactive species. Cracked sulfur is injected towards the substrate through a 1 cm diameter nozzle. Since the temperature of the cracked sulfur beam (here, 400 °C) is significantly above the condensation temperature of sulfur at mTorr pressures (50-100 °C) the fraction of sulfur that does not react with the growing film tends to stay in the gas phase long after its first collision. Thus, the sulfur partial pressure measured by an ion gauge and a remote OES setup located away from the direct sulfur beam is to be intended as a "background", or "diffuse" sulfur pressure. The growth-relevant partial pressure of sulfur is more difficult to estimate, and it strongly depends on the position of the film with respect to the 1 cm-wide nozzle from which sulfur is injected. This local pressure will generally be higher, or much higher, than the background pressure. The spatial distribution of the sulfur partial pressure is the enabling factor behind the sulfur composition gradients shown in Figure 1 of the main article.

The partial pressures of all gases can be monitored in-situ by a remote optical emission spectroscopy (OES) system (OPTIX, Gencoa). To produce the partial pressure time trace in Figure 1b of the main article, the remote plasma generator was run in pulsed DC mode with a 1 mA current setpoint. The partial pressures of the various gases were determined by the area of one of their OES peaks, with the following center wavelengths: 750.4 nm for Ar, 342.0 nm for PH, 656.7 nm for H, and a broad feature centered at 313.8 nm for S_2. The area of the peaks was converted to partial pressure by a calibration procedure, where known partial pressures of reactive gases were introduced in the chamber without any metal sources running while the corresponding OES peak areas were recorded.

To account for the reactivity of sulfur and PH_3, the pumps, seals, and all Cu, Ag, or Al-based metal parts are customized for corrosion resistance. Inert dilution gas is added at the turbomolecular and roughing pumping stages. PH_3 and H_2S exhaust gases are captured in a dry bed scrubber (Fab Support). At the time of the depositions presented in this paper, the chamber was also used to deposit Sb-, Ba-, and Zr-based compounds. Low-level Sb contamination (< 0.1%) in some of the Cu-P-S films was detected by XPS (Table S3). The incorporation of Sb is believed to arise from the high vapor pressure of Sb_2S_3. Key optoelectronic properties of Cu_3PS_4 (structure, absorption coefficient, PL spectra) are not correlated with the detection of Sb in the films.

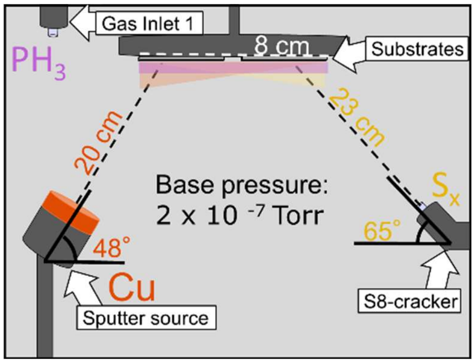

Figure S1. Schematic of the sputter chamber used for deposition with details on the geometry including angles and target to substrate distances.

Preparation: Substrates were mounted on the substrate holder covering an area of about 8x8 cm^2 before the start of the deposition. A combination of crystalline Si substrates (Siegert Wafer), fused silica substrates (Siegert Wafer) and soda-lime glass substrates (Avantor) were used for the depositions. The substrate holder was mounted face down above the sputter sources in the chamber. The base pressure of the sputter chamber before the deposition was below 2 x 10^{-7} Torr. Zone 1 of the S-cracker was heated 3 h before the deposition to ensure a stable temperature in the effusion cell. Zone 2 and Zone 3 were heated up 1 h before the deposition. The background partial pressure of sulfur was adjusted to the desired value by tuning the pulsed valve's duty cycle to achieve the desired pressure, as measured with an ion gauge without flowing additional gases. The substrate was heated up to 465 °C with a ramp rate of 20 °C/min using infrared lamps and left for 10 minutes to stabilize. The temperature of the substrate holder was determined by a calibration curve measured in a control experiment at the same pressure used in the deposition process. The calibration curve was obtained by simultaneously measuring the temperature in the lamp housing (used to control the heater) and the temperature measured at the substrate holder with an auxiliary thermocouple.

Synthesis: The precursors used for thin-film deposition were a 2-inch Cu target (Testbourne, 99.999%), PH_3 (Linde, 10% PH_3 99.9998% in Ar 99.9997%) and sulfur pellets (Thermo Scientific, 99.999%). During the deposition the pressure was controlled by adjusting the opening of the throttle valve in front of the turbo pump. To achieve the desired partial pressure of PH_3 a constant flow of 35 sccm 10% PH_3 in Ar and 100 sccm of Ar were supplied from the reactive and inert gas inlet, respectively. The three zones of the S-cracker were kept at 115±5 °C in Zone 1, 250 °C in Zone 2 and 400 °C in Zone 3. The valve "on" time and valve frequency were adjusted respectively between 20 to 50 ms and 8 to 10 Hz to achieve a stable sulfur partial pressure during deposition. The background sulfur vapor pressure was measured before deposition to be between 0.2 and 0.5 mTorr according to an ion gauge, with consistent values obtained by remote OES.

Cooldown: After the deposition the sputter target was ramped down completely, and the substrate heater was ramped down at a rate of 20 °C/min. To avoid re-evaporation of the volatile P and S species from the films, the S-cracker and gases were kept running at the same rates as during the deposition with the substrate shutter open. When the temperature at the substrate holder reached 195 °C, the reactive gases and the S-cracker were shut off. To accelerate the cool-down of the substrates and of the S-cracker, the pressure in the chamber was increased by filling it with additional Ar. The samples were taken out of the deposition chamber after they reached room temperature (25 °C) and moved into the attached N_2-filled glovebox without exposure to air. The glovebox is actively purified by circulating N_2 through oxygen, moisture, and organic solvent purifiers. Typical contamination levels are 0.1-1 ppm O_2, less than 0.1 ppm H_2O, and less than 0.1 ppm solvents.

Samples and Substrates: To account for the specific requirements of the characterization techniques used in this work, Cu_3PS_4 was deposited on three substrate types. Conductive Si substrates were used for electron-based characterization (SEM, EDX, XPS, UPS) to avoid charging. Transparent fused silica and soda-lime glass substrates were chosen for optical characterization. All samples were characterized by XRD to confirm that the same single-phase Cu_3PS_4 was deposited. EDX was used to verify the composition and SEM imaging was performed to ensure a similar morphology across different substrate materials and deposition runs. For clarity, the substrate material and deposition run of the sample used for each measurement technique is listed in Table S1 and Table S2.

Table S1. Information on the substrate material and deposition run for every measurement shown in this work

	**Material**	**Deposition run**
**Fig 1 EDX**	Si	C
**Fig 2 XPS**	Si	A
**Fig 3 XRD**	Fused silica	A
**Fig 3 Raman**	Si	A
**Fig 3 SEM**	Si	A
**Fig 4 ternary plot**	Fused silica and Si	A
**Fig 4 Photo**	Soda-lime glass	B
**Fig 5 Photo**	Soda-lime glass	B
**Fig 5 XRD (2 months in air)**	Si	D

**Fig 5 XRD (rest)**	Soda-lime glass	B
**Fig 6 PL**	Soda-lime glass	B
**Fig 6 absorption coefficient**	Soda-lime glass	B
**Fig 7 XPS**	Si	A
**Fig 7 UPS**	Si	A
**Fig S2**	Fused silica	A
**Fig S3**	Fused silica	A
**Fig S4**	Si	D (465°C), E (375°C), F (RT)
**Fig S5**	Si	D
**Fig S6**	Soda-lime glass	B
**Fig S7**	Si	A
**Fig S9**	Soda-lime glass	B
**Tab S3**	Si	A

Table S2. Information about the samples used in this work, their substrate composition and which measurements have been done on then

**Sample**	**Material**	**measurements**
**A1**	Fused silica	XRD (Fig 3, Fig S2, Fig S3) EDX (Fig 4)
**A2**	Si	XPS (Fig 2, Fig 7, Fig S7, Tab S3), Raman (Fig 3), SEM(Fig 3), EDX (Fig 4) UPS (Fig 7)
**B**	Soda-lime glass	Photo (Fig 4, Fig 5, Fig S6), XRD (Fig 5), PL (Fig 6) absorption coefficient (Fig 6, Fig S9)
**C**	Si	EDX (Fig 1)
**D**	Si	XRD (Fig 5 2 months in air), XRD(Fig S4)
**E**	Si	XRD(Fig S4)
**F**	Si	XRD(Fig S4)

EDX: The EDX measurements were done in an SEM at an acceleration voltage of 15 keV, a working distance of 10 mm and a measurement area of 130 µm^2. A typical beam current was 2 nA, resulting in a time-averaged current density of 1.5 mA/cm^2. Thus, the power density of the electron beam incident on the sample is about 20 W/cm^2. For the composition determination the K_α lines of S and P and the L_α line of Cu were used.

The data treatment was done in the AZtec software (Oxford Instruments) using its built-in database of standards and the add-on LayerProbe module to differentiate between the thin-film layer (assumed to be ideally flat and continuous) and the substrate. The thickness d of the film was deduced from the mass thickness ρd output from the LayerProbe model, with a film density ρ of 4.33 g/cm^3. We performed control experiments on various thin-film compounds with known compositions and different thicknesses to determine the origin and magnitude of the main sources of error in the best-fit composition calculated by LayerProbe. We found two main sources of composition error. The first is a material-dependent systematic error caused by the inherent inaccuracy of a general-purpose set of standards. This error is in the range of ± 1% relative. The second error is a film thickness-dependent error, arising from changes in the relative intensity of peaks from the film and from the substrate, significantly altering the spectra the model is fitted against. While the thickness-dependent error is vastly improved compared to standard quantitative EDX software for bulk samples owing to explicit modeling of a multi-layer stack, it is still about ± 1% relative in the thickness range relevant for this study. Thus, we can expect a total error of $(1^2 + 1^2)^{1/2} \approx \pm$ 1.4% relative, and therefore a 50% sulfur atomic composition is to be intended as (50 ± 0.7) %. The large surface roughness makes thickness determination by EDX less reliable, but it does not add a significant error to the quantified composition.

SEM: The SEM images were recoded with an acceleration voltage of 3 kV and a working distance of 8 mm using the SE2 detector.

XPS/UPS: XPS and UPS were conducted in a Thermo Fischer Nexsa surface analysis system with a base pressure in the 10^{-8} Torr range. The Cu_3PS_4 film on a conductive silicon substrate was in electrical contact with the spectrometer, and the binding energy scale was calibrated with standard metallic samples. No evidence of sample charging was found during the XPS and UPS scans. For the XPS survey scan, a pass energy of 200 eV, a step size of 1 eV and 30 scans were chosen. For the core level

scans, a pass energy of 50 eV and a step size of 0.1 eV were chosen together with an appropriate number of scans to resolve the peaks. The Avantage software (Thermo Fischer) was used for peak fitting and for quantification of the surface elemental composition. For the peak fitting the "Smart" background option of Avantage was used together with the photoionization cross section values from Thermo Fisher's own "SF AlThermo1" set. For doublet peaks, fixed height ratios and equal FWHM were enforced. The surface elemental composition was determined from the areas of the Cu $2p_{3/2}$ (SF: 18.147), P $2p_{3/2}$ (SF: 0.896), S $2p_{3/2}$ (1.245), O 1s (2.881), Sb $3d_{5/2}$ (SF: 23.500) and C 1s (SF: 1.000) peaks in the core level scans, with an expected accuracy of about ± 5% relative for the elemental composition.[1] A monochromated Al-Kα source was used for all XPS measurements. The measurement spot size was 400 µm for XPS and about 1.5 mm for UPS. The UPS measurement was conducted with He-I source with a pass energy of 2 eV, step size of 0.05 eV and 15 scans.

XRD: The Rigaku SmartLab system used for XRD measurements was equipped with a high-power 9 kW Rotating Anode Cu Kα source and a HyPix-3000 2D detector, in parallel beam θ/2θ geometry without $K\alpha_2$ or Kβ filters. A CBO-f focusing element coupled with 0.5mm length limiting slit were used as incident optics yielding a spot size of approximately 0.5mm in diameter. No receiving optics were used, and the detector to sample distance was set to 200 mm. 2θ scans are calculated by integration over rings of constant 2θ of the X-ray intensity recorded of the 2D detector.

LeBail refinement was performed in FullProf with crystal symmetry *Pmn21*, on the raw integrated intensity data. The pattern was refined with a background consisting of linear interpolation of manually defined points, and by fitting the peaks with Pseudo-Voigt profiles. The refined lattice parameters *a* = 7.2907(±0.0005), *b* = 6.3019(±0.0004), *c* = 6.0617(±0.0003) were obtained with fit quality indicators Rp = 27.0, Rwp = 18.0, Re = 25.3., However, the standard errors calculated by the fit are typically underestimated for thin film sample, due to, e.g., vertical sample misalignment, heterogenous strain, and significant peak broadening. Therefore, we give the lattice parameters with three-digit precision in the main article. There are four post-1950 entries for bulk enargite Cu_3PS_4 in the ICSD database measured under ambient conditions. The average and standard deviation of the lattice parameters in these entries are *a* = 7.288(±0.008), *b* = 6.330(±0.008), *c* = 6.075(±0.004).

Raman: The Raman measurements were done with a 785 nm laser and a 20x objective. The spot size for this measurement is 2.5 µm in diameter and the light intensity is 1000 W/cm^2 (10 000 sun equivalents).

Optical: The Agilent Cary 7000 Universal Measurement Spectrophotometer (UMS) instrument was equipped with the Diffuse Reflectance Accessory (DRA) integrating sphere, and the Small Spot Kit add-on, The spot size was measured to be approx. 1 mm in diameter. The absorption coefficient α was deduced from the total (i.e. diffuse and specular) UV-vis-IR reflection (R) and transmission (T) spectra recorded at quasi-normal (8°) and normal (0°) incidence, respectively, and from film thickness d (deduced from EDX) using the single reflection approximation, without substrate subtraction:

$$\alpha = -\frac{1}{d}\ln\left(\frac{T}{1-R}\right) \quad (1)$$

PL: The PL spectra were recorded using a custom setup consisting of the following parts. A 50x objective (Numerical Aperture of 0.6) objective directs the 405 nm blue laser (Matchbox) onto the sample. The emitted light is filtered by a long pass filter dichroic mirror at 420 nm. The spot size diameter and photon intensity has been estimated to be 5 µm and 300 W/cm^2, respectively, which corresponds to 3 000 sun equivalents. All PL measurements were performed at room temperature.

Method to estimate experimental band gap by aligning the computed and experimental absorption spectra

We notice that the shapes of the computed and measured absorption spectra (Figure 6b in the main article) are very similar when multiplying the whole computed α by a factor 3 (Figure S9). Thus, we apply a methodology similar to the one that was used in Figure 6c of the main article to determine the valence band position by a combination of a UPS measurement and a DOS calculation (Kraut method). The computed and measured absorption spectra coincide by shifting the computed α to 0.18 eV lower photon energy. Because the HSE06 direct gap of Cu_3PS_4 is 2.59 eV (indirect transitions are not included in the calculation), the experimental direct gap is estimated as 2.41 eV and the indirect gap as 2.35 eV. The factor 3 difference

between the calculated and measured absorption coefficient may be due to a combination of (i) high surface roughness, increasing the optical path in the film beyond the normal incidence plane-wave approximation, (ii) an ill-defined, possibly underestimated film thickness by the EDX measurement, also due to the large roughness, and (iii) the existence of additional absorption mechanisms not included in the calculation, such as indirect absorption[2] or excitonic effects[3].

Estimation of Cu oxidation state and charge state from an XPS Wagner plot

It is known that identification of the Cu charge state solely based on the Cu 2p core level binding energy is ambiguous because the peak ranges of materials with Cu in the 0 and +1 oxidation state overlap. Additional difficulties are that materials with +1 and +2 oxidation states often consist of a mixture of both species, and that Cu ions that are formally in the +1 oxidation state but have different actual charges are indistinguishable. Different advanced strategies have been discussed to gain insights on the chemical state of Cu in various compounds.[4] A widely adopted method to deconvolute these effects is the use of the Wagner or chemical state plot, visualizing the Cu $2p_{3/2}$ binding energy against the Cu $L_3M_{45}M_{45}$ Auger kinetic energy. It is known that Cu compounds with the formal oxidation state +1 exhibit a similar chemical shift and therefore similar Cu $2p_{3/2}$ binding energy position. The Auger kinetic energy position depends on the polarization of Cu after losing an electron in the photoemission process, and therefore on the covalency of the bonds, which is related to the actual charge localized on the Cu ion instead of to its formal oxidation state.[4]

Method to estimate valence band onset by aligning UPS spectrum and DOS calculation (Kraut method)

First, the HSE06-calculated valence band DOS of Cu_3PS_4 is convoluted with a gaussian function to match the instrumental broadening of the UPS measurement. Then, the DOS is shifted horizontally to align it to the 0-1 eV region of the UPS spectrum. In the UPS measurement, the Fermi level of the Cu_3PS_4 surface is equal to a binding energy of zero assuming good electrical contact between the sample and the spectrometer. In the DOS calculation, the VBM of Cu_3PS_4 is set to zero electron energy. Thus, the position of the VBM of the Cu_3PS_4 surface with respect to the Fermi level $(E_v - E_F)$ is equal to the shift applied to the DOS (here, –0.23 eV). The error bar of this VBM position determination is quoted as ± 0.02 eV.[5] The surface carrier concentration p can be derived by applying Fermi statistics to the effective density of states in the valence band N_v

$$p = N_v \frac{1}{\exp\left(\frac{E_F - E_v}{k_B T}\right) + 1} \quad (2)$$

Here, $(\boldsymbol{E_v} - \boldsymbol{E_F})$ is the quantity found above, and $\boldsymbol{N_v}$ is defined assuming quasi-parabolic bands at the VBM

$$N_v = 2\left(\frac{2m_{hDOS}k_B T}{h^2}\right)^{\frac{3}{2}} \quad (3)$$

Where $\boldsymbol{m_{hDOS}}$ is the density of states hole effective mass of Cu_3PS_4, which can be estimated from the geometric average $\boldsymbol{m_h}$ of the direction-resolved conductivity hole effective masses calculated with BoltzTraP2 (see the main article) and the number of valence band pockets $\boldsymbol{g_v}$.

$$\boldsymbol{m_{hDOS} = g_v^{\frac{2}{3}} m_h} \quad (\boldsymbol{4})$$

For Cu_3PS_4, the VBM at the Y point is within $\boldsymbol{k_B T}$ of another valence band pocket at the Γ point. Thus, we take $\boldsymbol{g_v = 2}$ and $\boldsymbol{m_h = 1.00\ m_0}$, yielding $\boldsymbol{N_v = 5 \times 10^{19}\ cm^{-3}}$ and $\boldsymbol{p = (6 \pm 4) \times 10^{15}\ cm^{-3}}$.

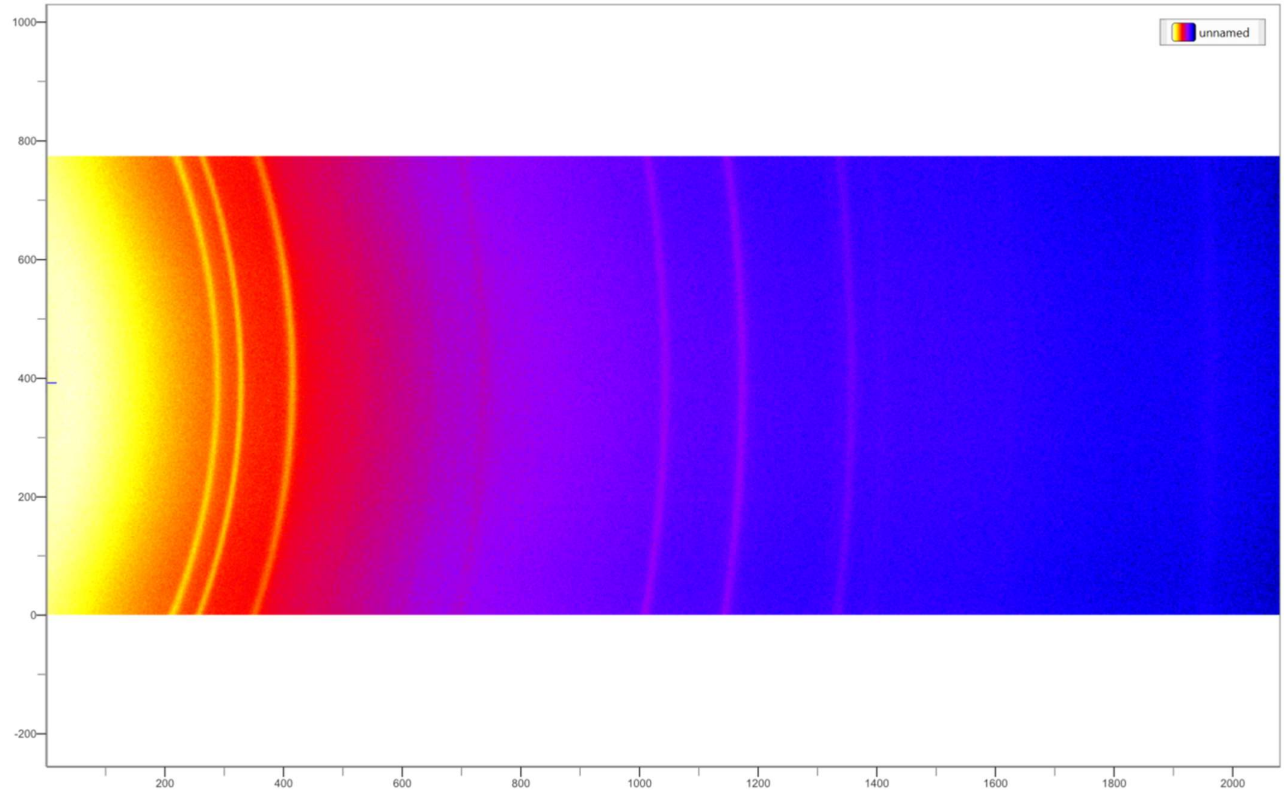

Figure S2. 2D diffraction image of the XRD measurement.

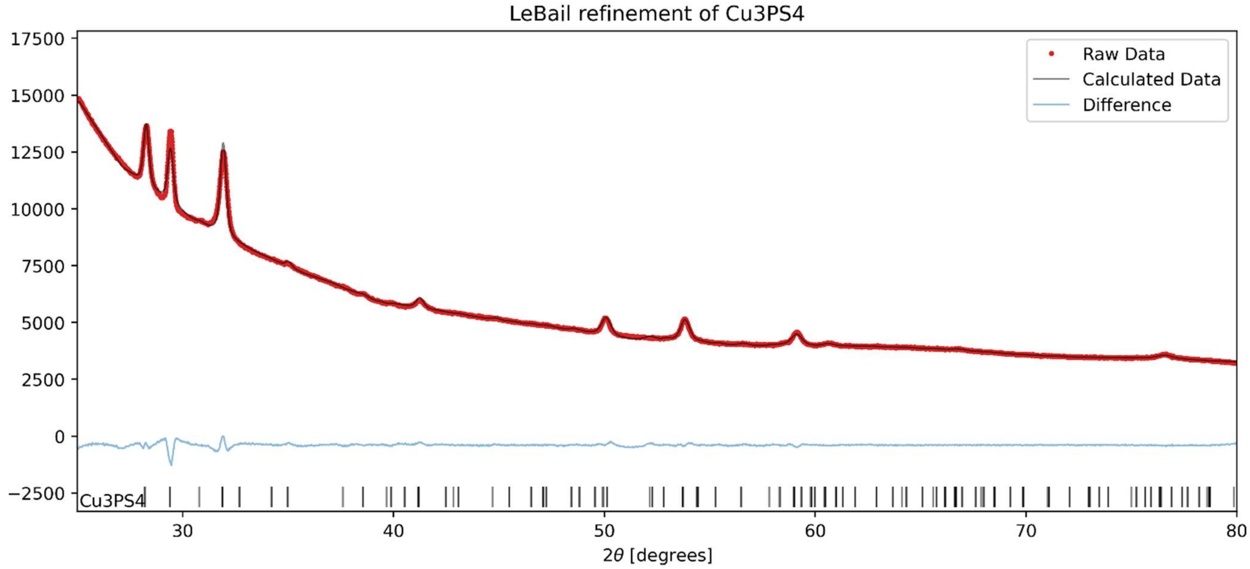

Figure S3. LeBail refinement of the XRD pattern of a Cu_3PS_4 film done in FullProf Suite.[6]

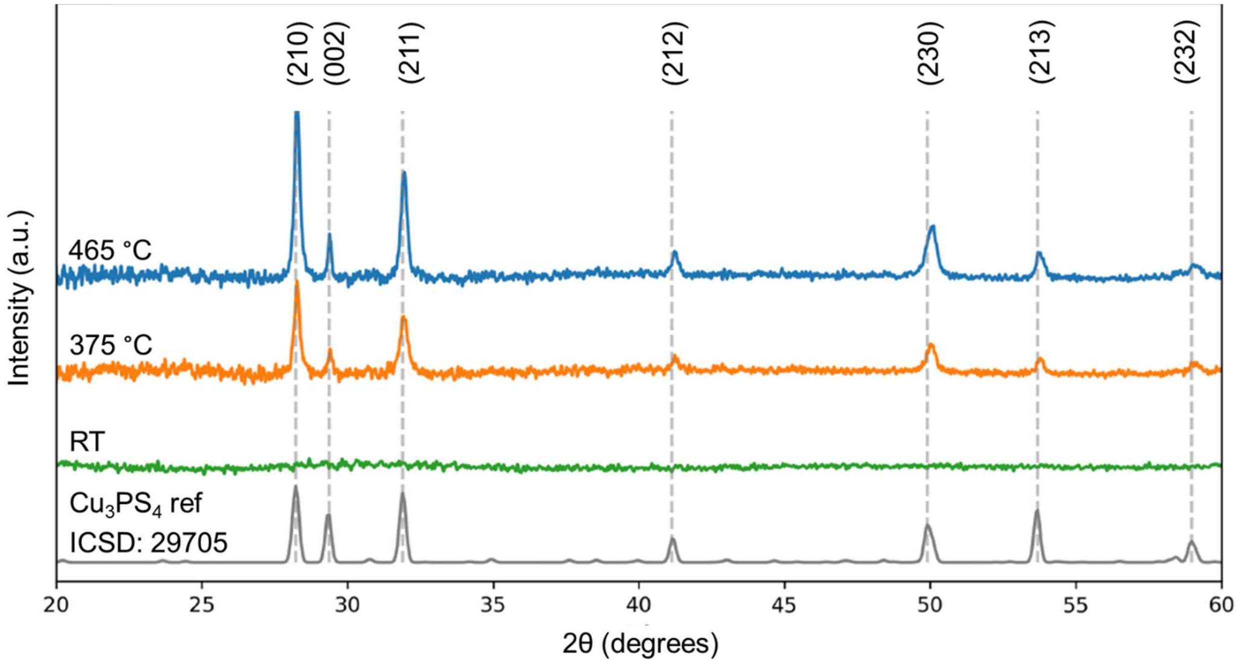

Figure S4. XRD patterns of films deposited at different temperatures (RT, 375 °C, 465 °C)

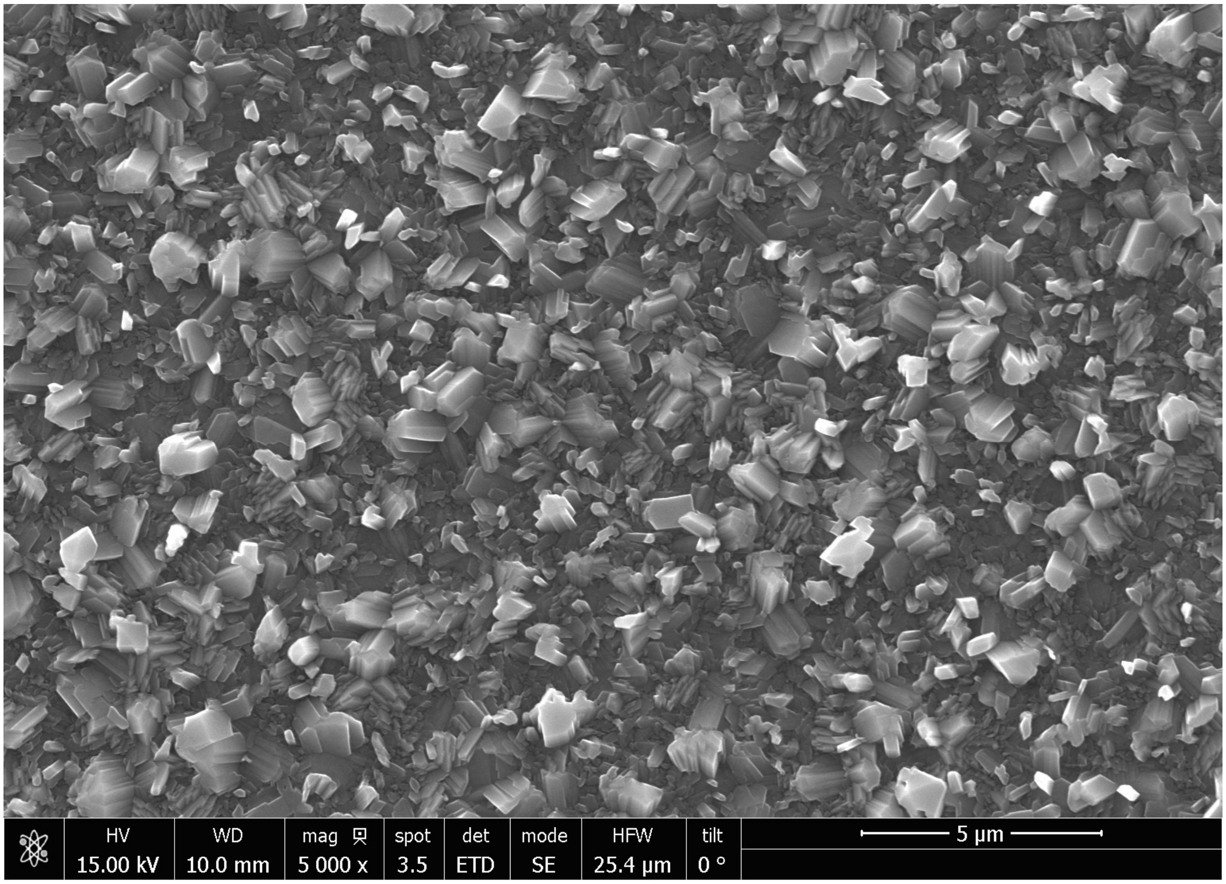

Figure S5. Lower magnification SEM micrograph of a Cu_3PS_4 thin film deposited at 465 °C.

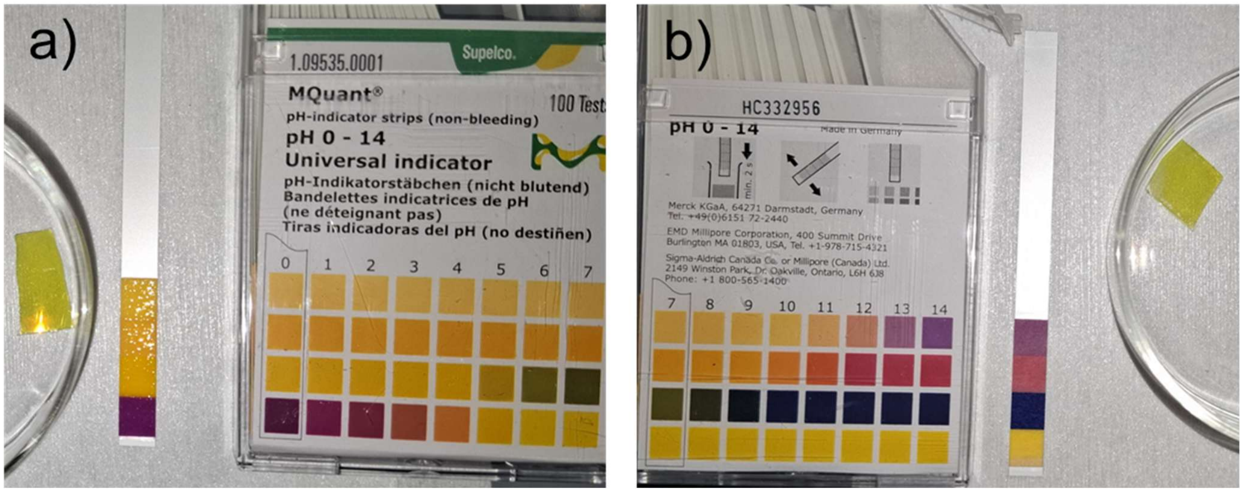

Figure S 6. Pictures from the stability tese with pH indicator for a) 1M HCl and b) 1M KOH

Table S33. Composition determined from XPS including all detected elements as well as the respective peak positions on a binding energy versus Fermi level scale. Values in parenthesis are on a kinetic energy scale. Peak positions in $CuInS_2$ and Cu_2ZnSnS_4 are from previous work. α' is the modified Auger parameter for Cu, defined as the sum of the binding energy of the Cu $2p_{3/2}$ peak and the kinetic energy of the Cu LMM Auger peak.

**Element**	**Cu**	**Cu auger**	**Cu**	**P**	**S**	**O**	**C**	**Sb**
**Peak type**	$2p_{3/2}$	LMM	α'	$2p_{3/2}$	$2p_{3/2}$	1s	1s	$3d_{5/2}$
**Atomic % composition**	27.47			9.21	40.91	4.16	18.19	0.06
**Peak position (eV)**	932.33	569.7 (916.9)	1849.2	131.63	162.06	532.68	284.54	529.89
**Peak position in $CuInS_2$ (eV)** [7]	932.2	(916.8)	1849.0		162.0			
**Peak position in Cu_2ZnSnS_4 (eV)**[8]	932.1	(916.9)	1849.0		161.8			

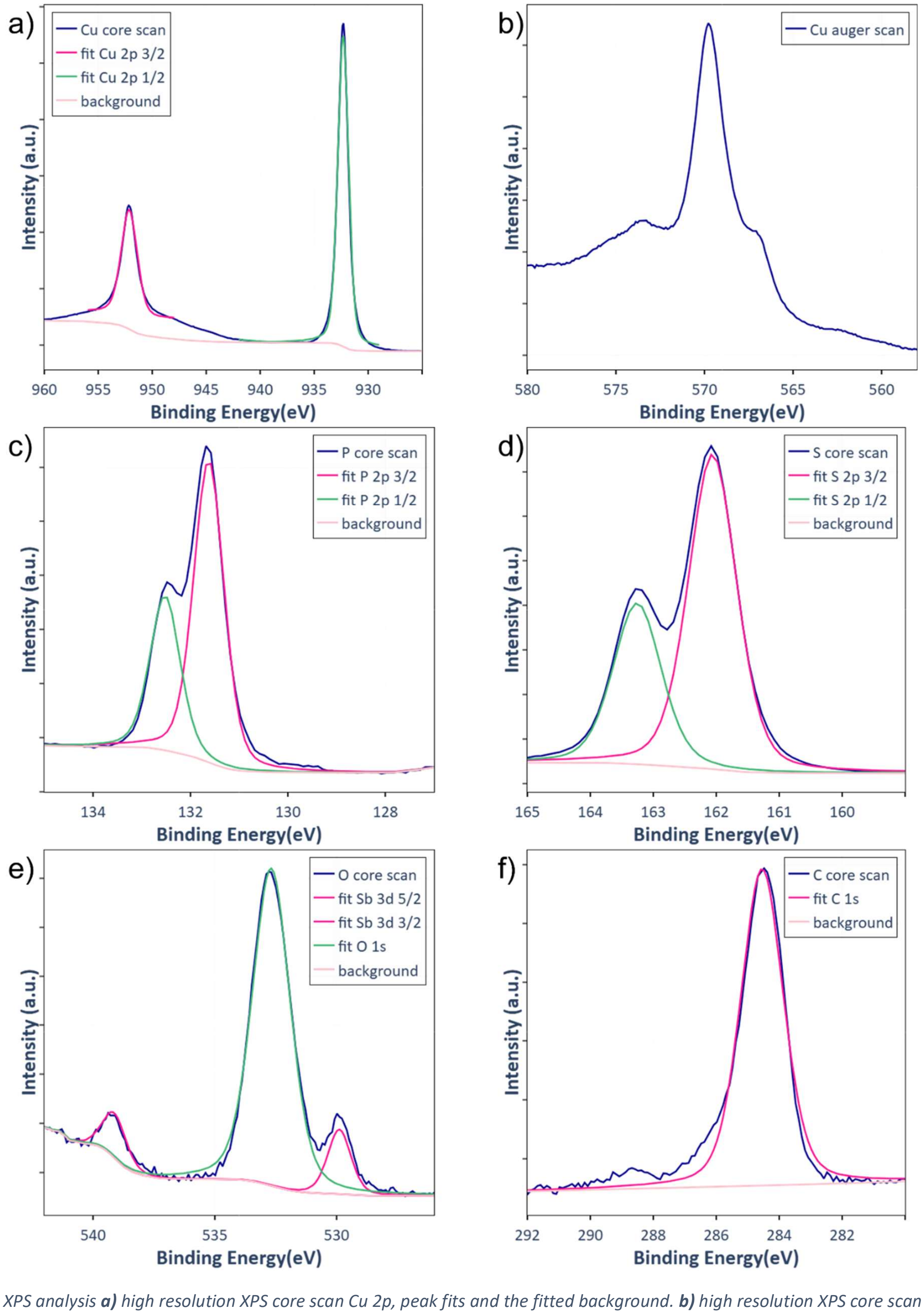

*Figure S7. XPS analysis **a)** high resolution XPS core scan Cu 2p, peak fits and the fitted background. **b)** high resolution XPS core scan of the Cu LMM region, **c)** high resolution XPS core scan P 2p, peak fits and the fitted background. **d)** high resolution XPS core scan S 2p, peak fits and the fitted background. **e)** high resolution XPS core scan O 1s and Sb 3d, peak fits and the fitted background. **c)** high resolution XPS core scan C 1s, peak fits and the fitted background.*

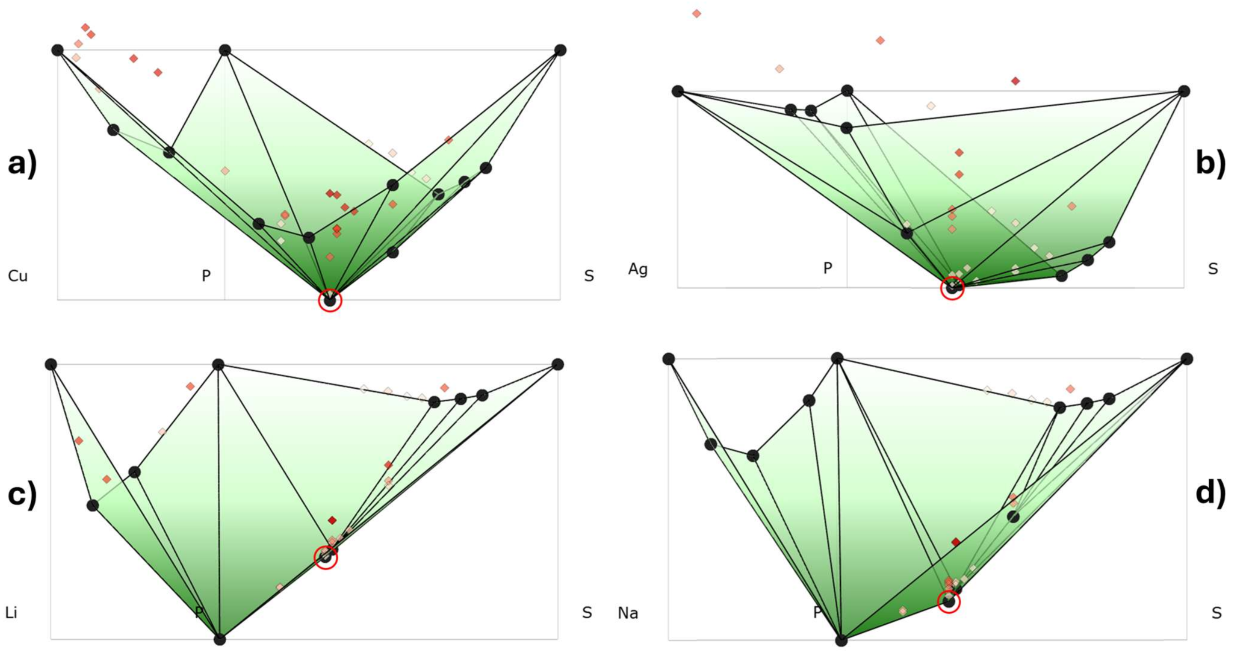

Figure S8. PBESol convex hulls for the M-P-S systems with M as the +1 oxidation state metals Cu (a), Ag (b), Li (c) and Na (d). The red circle indicates the M_3PS_4 composition. It is only for the case of the Cu-P-S system that the M_3PS_4 phase results in substantial energy lowering.

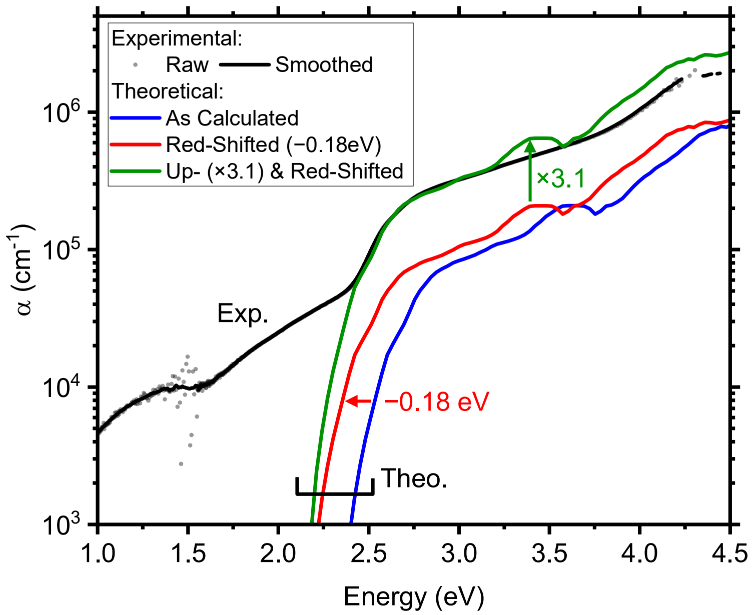

Figure S9. Comparison of the measured absorption coefficient (black) and the as calculated absorption coefficient (blue) plotted on a photon energy scale. The red curve corresponds to the HSE06-calculated absorption coefficient red-shifted by 0.18 eV towards lower energy, as compared to the as calculated absorption coefficient. In the green curve, the red-shifted absorption coefficient is also scaled by a factor of 3.1, to match the experimental absorption coefficient onset. This is a method to estimate the direct band gap in the Cu_3PS_4 film as the HSE06 direct band gap (2.59 eV) minus the 0.18 eV shift, yielding a value of 2.41 eV.

Supplementary references

S1 G. Greczynski and L. Hultman, A step-by-step guide to perform x-ray photoelectron spectroscopy, J. Appl. Phys., 2022, 132, 011101.

S2 J. Willis, I. Bravić, R. R. Schnepf, K. N. Heinselman, B. Monserrat, T. Unold, A. Zakutayev, D. O. Scanlon and A. Crovetto, Prediction and realisation of high mobility and degenerate p-type conductivity in CaCuP thin films, Chem. Sci., 2022, 13, 5872–5883.

S3 J. Vidal, S. Lany, M. D'Avezac, A. Zunger, A. Zakutayev, J. Francis and J. Tate, Band-structure, optical properties, and defect physics of the photovoltaic semiconductor SnS, Appl. Phys. Lett., 2012, 100, 032104.

S4 M. C. Biesinger, Advanced analysis of copper X-ray photoelectron spectra, Surf. Interface Anal., 2017, 49, 1325–1334.

S5 E. A. Kraut, R. W. Grant, J. R. Waldrop and S. P. Kowalczyk, Precise Determination of the Valence-Band Edge in X-Ray Photoemission Spectra: Application to Measurement of Semiconductor Interface Potentials, Phys. Rev. Lett., 1980, 44, 1620–1623.

S6 O. Arcelus, J. Rodríguez-Carvajal, N. A. Katcho, M. Reynaud, A. P. Black, D. Chatzogiannakis, C. Frontera, J. Serrano-Sevillano, M. Ismail, J. Carrasco, F. Fauth, M. R. Palacin and M. Casas-Cabanas, FullProfAPP : a graphical user interface for the streamlined automation of powder diffraction data analysis, J. Appl. Crystallogr., 2024, 57, 1676–1690.

S7 R. Scheer and H. J. Lewerenz, Photoemission study of evaporated CuInS2 thin films. II. Electronic surface structure, J. Vac. Sci. Technol. Vac. Surf. Films, 1994, 12, 56–60.

S8 L. Köhler, doctoral thesis, BTU Cottbus - Senftenberg, 2017.
